# Remote Lifestyle Intervention to Reduce Postpartum Weight Retention: Protocol for a Community-Engaged Hybrid Type I Effectiveness-Implementation Randomized Controlled Trial

**DOI:** 10.2196/62847

**Published:** 2025-01-07

**Authors:** Lindsay M Martin, Christine D McKinney, Lia Escobar Acosta, Janelle W Coughlin, Noelene K Jeffers, Alexandra Solano-Umaña, Kathryn A Carson, Nae-Yuh Wang, Wendy L Bennett, Kelly M Bower

**Affiliations:** 1 Division of General Internal Medicine Department of Medicine Johns Hopkins School of Medicine Baltimore, MD United States; 2 Johns Hopkins School of Nursing Baltimore, MD United States; 3 Department of Psychiatry Johns Hopkins School of Medicine Baltimore, MD United States; 4 The Lourie Center Head Start Program Adventist HealthCare Rockville, MD United States; 5 Department of Epidemiology Johns Hopkins Bloomberg School of Public Health Baltimore, MD United States

**Keywords:** pregnancy, obesity, postpartum weight retention, remote lifestyle intervention, home visiting, mobile health, mHealth app, community-engaged research, implementation science, health disparities, maternal health

## Abstract

**Background:**

Maternal obesity is associated with significant racial disparities. People who identify as non-Hispanic Black and Latinx are at the highest risk related adverse short- and long-term health outcomes (eg, hypertension in pregnancy and postpartum weight retention). Remote lifestyle interventions delivered during and after pregnancy hold promise for supporting healthy weight outcomes; however, few are tested in groups of people who self-identify as non-Hispanic Black and Latinx or address the neighborhood-level and psychosocial factors driving maternal health disparities. Implementing remote lifestyle interventions within community-based programs that serve birthing people may optimize trust and engagement, promote scalability and sustainability, and have the broadest public health impact.

**Objective:**

The goal of this trial is to test the effectiveness of a culturally adapted remote lifestyle intervention (Healthy for Two–Home Visiting) implemented within home visiting compared to usual home visiting services on postpartum weight retention among pregnant or postpartum individuals, in particular those who identify as non-Hispanic Black and Latinx. Facilitators and barriers to implementation of the intervention within home visiting will be examined.

**Methods:**

We describe the rationale and protocol for this hybrid type I effectiveness-implementation randomized controlled trial. In this paper, we highlight the community-engaged approach and trial design features that enable the implementation of the intervention within home visiting and demonstrate its applicability to the target population. Participants will be 360 pregnant individuals with overweight or obesity enrolled between 20 and 33 weeks of gestation and randomized 1:1 to Healthy for Two–Home Visiting or usual home visiting services. The primary outcome is weight retention at 6 months post partum, calculated as 6-month postpartum weight minus earliest pregnancy weight (≤18 wk of gestation). The measures of implementation include intervention feasibility, acceptability, reach, adoption, and fidelity. Throughout the paper, we highlight the community input used to improve intervention effectiveness and study implementation and as a strategy to promote maternal health equity.

**Results:**

This study was funded in June 2021, and recruitment began in April 2023. As of November 2024, we enrolled 90 participants. Data collection to assess the intervention’s effectiveness is expected to end in June 2026. Implementation evaluation is expected to conclude in December 2026.

**Conclusions:**

This hybrid type I effectiveness-implementation randomized controlled trial integrates a culturally adapted remote lifestyle intervention into early home visiting services to examine its effectiveness on postpartum weight retention compared to usual home visiting. We anticipate that the study results will enable an understanding of the drivers of successful implementation within a community-based setting to maximize the future sustainability and dissemination of a strategy for reducing long-term obesity and other maternal health disparities.

**Trial Registration:**

Clinicaltrials.gov NCT05619705; https://clinicaltrials.gov/study/NCT05619705

**International Registered Report Identifier (IRRID):**

DERR1-10.2196/62847

## Introduction

### Background

Maternal obesity is a persistent public health concern, with widening racial and ethnic inequities [1-3]. In the United States, 57% of women who self-identify as non-Hispanic Black and 47% of women who self-identify as Latinx, Hispanic, or of Spanish origin (hereinafter referred to as Latinx) have obese status compared to 38% of people who identify as non-Hispanic White [4]. Nearly 50% of pregnant people who identify as non-Hispanic Black or Latinx exceed the recommended guidelines for gestational weight gain (GWG), contributing to adverse maternal and infant health outcomes (eg, hypertension in pregnancy, preterm birth, and maternal mortality) [5-8], as well as an estimated economic impact of up to US $32 billion from conception through the offspring’s first 5 years of life [9]. It is imperative to focus public health prevention efforts on non-Hispanic Black and Latinx pregnant individuals who are most susceptible to worsening obesity (ie, postpartum weight retention [PPWR]) [10-13] and other long-term health problems, including cardiovascular disease [14-17]. Pregnancy offers an opportunity to initiate healthy behaviors that limit GWG and its associated health risks because individuals are motivated to have a healthy baby [18]. This ideal window for health promotion extends to the period after birth when it is critical to sustain healthy changes and improve care transitions, especially among individuals with known barriers to health care access and quality [19]. These individuals have increased exposure to negative social determinants of health (eg, environmental, financial, cultural, and linguistic barriers; racism; limited health literacy; and inadequate insurance coverage), which impacts postpartum visit attendance [20,21] and further exacerbates health risk [22-24].

Counseling and lifestyle interventions during and after pregnancy are a recommended and well-established strategy for limiting GWG [25-28] and reducing PPWR [29-32], and their implementation is being tested in real-world settings; for example, our team is testing a remote health coaching intervention to limit GWG integrated into prenatal care clinics [33,34]. However, there are several evidence gaps. First, few interventions have been tested in racial and ethnic minority groups [32,35], with especially low representation of Latinx individuals [36]. Second, few interventions have been implemented and tested in community-based settings where pregnant and postpartum individuals considered high risk access safety net services. Finally, interventions that address health-constraining social factors that contribute to disparities in maternal health outcomes are limited [31,37,38].

Importantly, implementing effective remote lifestyle interventions within community-based programs that pregnant individuals access and trust may optimize their benefits, promote scalability and sustainability, and have the broadest public health impact. Home visiting is an evidence-based public health strategy targeting pregnant individuals considered high risk and families with children aged up to 5 years. Home visitors provide health education, promote positive parenting and early learning, and link families with needed community resources and social support [39]. Early home visiting has been shown to prevent child abuse and neglect, improve maternal and child health, enhance family socioeconomic status, and promote child development and school readiness [40]. Early home visiting is an ideal setting for delivering lifestyle interventions for pregnant and postpartum individuals because home visitors are uniquely positioned to address social and environmental factors impacting health behavior (eg, neighborhood food availability and walkability) [39]. A recent randomized trial testing a lifestyle intervention embedded in early home visiting services showed lower GWG and PPWR up to 12 months, greater achievement of 5% weight loss, smaller waist circumference, and reduced sugar intake at 12 and 24 months [41], as well as greater success in reducing access to sugar-sweetened beverages in the home up to 24 months [41,42].

### Objectives

The goals of this paper are to (1) describe the design of this hybrid type I effectiveness-implementation randomized controlled trial testing the effectiveness of the Healthy for Two–Home Visiting (H42-HV) remote lifestyle intervention integrated into home visiting compared to usual home visiting services on PPWR among pregnant and postpartum individuals; (2) highlight the design features of this trial that enable its implementation within home visiting and the applicability of the intervention to the target population, in particular those who identify as Latinx and non-Hispanic Black; and (3) highlight our application of a community-engaged approach to the conceptualization and design of the study to improve intervention effectiveness and study implementation and as a strategy to promote maternal health equity.

## Methods

### Study Design, Aims, and Hypothesis

We designed this hybrid type I effectiveness-implementation randomized controlled trial to test the effect of the H42-HV lifestyle intervention integrated into home visiting from mid- to late pregnancy (20-33 wk) through 6 months post partum, compared to usual home visiting services, among pregnant and postpartum individuals with overweight or obesity. The primary outcome is PPWR calculated as 6-month postpartum weight minus prepregnancy (≤18 wk of gestation) weight. Additional measures of effectiveness include GWG and maternal health behaviors, wellness, and health care use. Our main hypothesis is that participants in the H42-HV arm will have lower PPWR than those in the usual home visiting services arm.

Hybrid type I effectiveness-implementation trials assess the primary outcome of clinical effectiveness and evaluate implementation strategies of the intervention as secondary outcomes to better understand facilitators and barriers to real-world dissemination. This hybrid approach could efficiently and in a timely fashion inform the pathways from translation of evidence into practice upon establishing the effectiveness of the intervention, guide future sustainability efforts, and facilitate greater subsequent public health impact [43,44]. To this end, the study will also examine home visiting organizational factors that could impact the implementation of the intervention. We will use the practical, robust implementation and sustainability model (PRISM) framework [45] and domains from the Consolidated Framework for Implementation Research (CFIR) [46] to assess intervention feasibility, acceptability, reach, adoption, and fidelity.

The protocol has been registered with ClinicalTrials.gov (NCT05619705).

### Application of a Community-Engaged Approach

We used a community-engaged research approach to inform the conceptualization and design of the study, including the adaptation of the H42-HV intervention and its integration into early home visiting services. On the basis of the continuum of community engagement in research [47]*,* our level of engagement is best characterized as community participation because the community was actively engaged with a defined role in all stages of the research process. Prior studies clearly demonstrate the importance of early and sustained stakeholder involvement to develop and implement remote health interventions for underserved populations [48-50]. The study principal investigators (WLB and KMB) engaged home visiting stakeholders while developing the proposal and, once funded, used a variety of strategies to establish and sustain 2-way engagement, communication, and information sharing. All aspects of the study were enhanced by feedback from a diverse group of stakeholders who serve individuals identifying as Latinx or non-Hispanic Black, including regional and state leaders in home visiting and participating home visiting program managers and home visitors. Stakeholders also included current or recently pregnant individuals who identify as Latinx or non-Hispanic Black and participate in home visiting services.

During the conceptualization phase, we met with state and program leaders to gather information about the relevance of the intervention and its alignment with state and program public health priorities. We also explored the feasibility and acceptability of implementing the intervention within the home visiting setting. In the planning phase of the study, we established a translation and cultural adaptation team of primarily native Spanish-speaking maternal and child health professionals (ie, dietitian, midwife, and nurse) and health professional students (ie, those studying nursing and medicine) to translate and adapt the H42-HV intervention for Spanish-speaking individuals (the adapted version is called Sanos los Dos).

**Figure 1:**
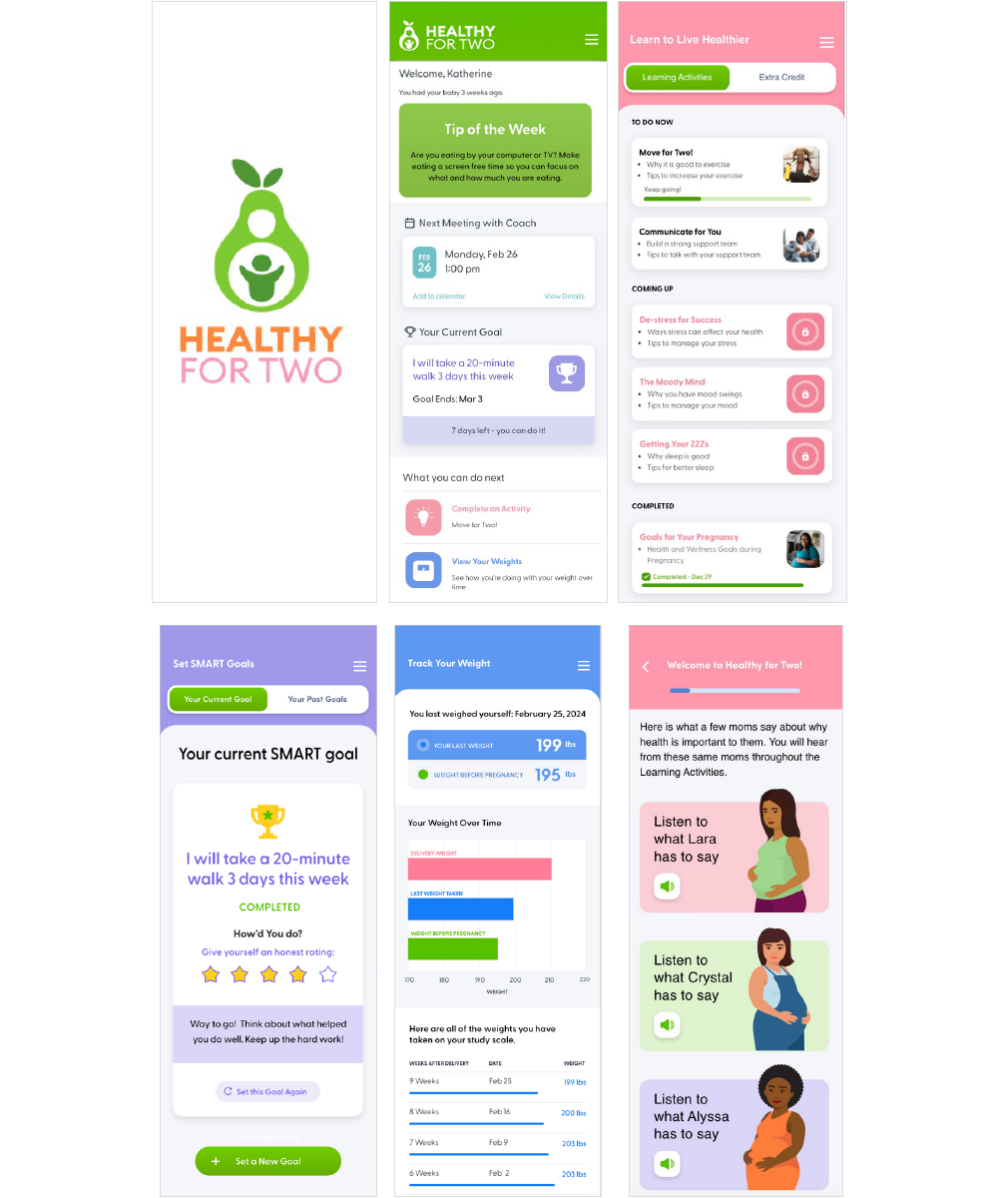
Healthy for Two mobile health app.

Once funded, we established a coordinating council with home visitors, leaders from participating programs, and Spanish- and English-speaking community members. Regular meetings with the coordinating council informed all aspects of the study protocol as well as implementation measures, recruitment processes, intervention adaptation, and safety protocols. We asked for specific feedback about the referral process, recruitment materials (flyers and videos), intervention approach and messaging, cultural adaptability, and community resource needs through semistructured one-on-one interviews (6 with home visiting program leaders and 7 with coordinating council members). We performed end-user testing of the H42 mobile health (mHealth) app (Figure 1). We conducted 6 interviews with parents and 2 with home visitors, applying a process known to impact the usability and engagement of culturally adapted digital health tools [49,51].

Overall, the feedback highlighted facilitators and barriers to the integration of the H42-HV intervention into home visiting programs and identified strategies for recruitment, adaptations to meet the language and cultural needs of individuals who identify as Latinx and non-Hispanic Black, and effective coordination between the home visitor and health coach. We describe how we addressed feedback from the coordinating council and the additional stakeholders in each of the following subsections.

### Home Visiting Programs and Setting

In the formative phase of the trial, we engaged with 7 home visiting programs from across 5 counties in Maryland, United States, that serve predominantly pregnant and postpartum individuals who identify as Latinx or non-Hispanic Black, speak English and Spanish, and have low incomes and literacy levels. Once we launched recruitment, we invited additional early home visiting programs to refer participants to be screened and enrolled in the study. We did not limit ourselves to a particular model of home visiting and included evidence-based and non–evidence-based models [39]; for example, the partnering home visiting models include but are not limited to Healthy Families America, Healthy Start, Nurse Family Partnership, and Babies Born Healthy. Depending on the model, home visitors are either nurses or paraprofessionals. Participating home visiting models enroll families in early pregnancy and follow them 6 months to 5 years post partum, but the frequency and intensity of home visits vary by model.

### Participant Eligibility

As this is an effectiveness trial, we apply the broadest eligibility criteria to enhance generalizability [43,44,52]: age ≥18 years, singleton pregnancy between 20 and 33 weeks of gestation, and planning to enroll in home visiting services at 1 of the study’s participating sites. We are focusing this study on individuals who are overweight or obese (BMI ≥25 kg/m^2^) before pregnancy as they are at the highest risk for future cardiometabolic disease [53], and we are excluding conditions that may impact an individual’s ability to medically or physically participate in the intervention if randomized to that arm (eg, advised not to exercise by provider or diagnosed with type 1 diabetes). Textbox 1 presents additional eligibility criteria.

**Eligibility criteria.**

**Inclusion criteria**

- Age ≥18 y
- 20-33 wk of gestation
- Prepregnancy BMI ≥25 kg/m^2^ (calculated based on self-reported prepregnancy height and weight)
- Able to provide informed consent
- English or Spanish speaking
- Intention to enroll in early home visiting services at a participating site
- Ability to complete telephone-assisted screening and electronic consent

**Exclusion criteria**

- Diagnosed with type 1 diabetes
- Pregnant with multiple fetuses
- Advised not to engage in exercise by medical provider
- Not cleared by the study’s clinicians or home visiting program staff
- Planning to relocate outside of Maryland in the next year
- Active substance abuse (except marijuana)
- Psychiatric or substance use–related hospitalization in the past year
- Active eating disorder

Evidence shows that starting an intervention early in pregnancy has the greatest impact on pregnancy outcomes and GWG [54,55]. However, many home visiting programs rely on several steps to occur before services can begin, that is, entry in prenatal care, referrals from clinic, screening by outside agency for eligibility, and outreach by home visiting program. In response to input from participating home visiting programs, we selected a broad enrollment window during pregnancy (20-33 wk of gestation) and will continue intervention delivery through 6 months post partum. Given state and program leader feedback about the potential for home visiting enrollment in late pregnancy, we selected the primary outcome as return to prepregnancy weight or below because PPWR is a risk factor for future obesity.

### Screening and Recruitment

**Figure 2:**
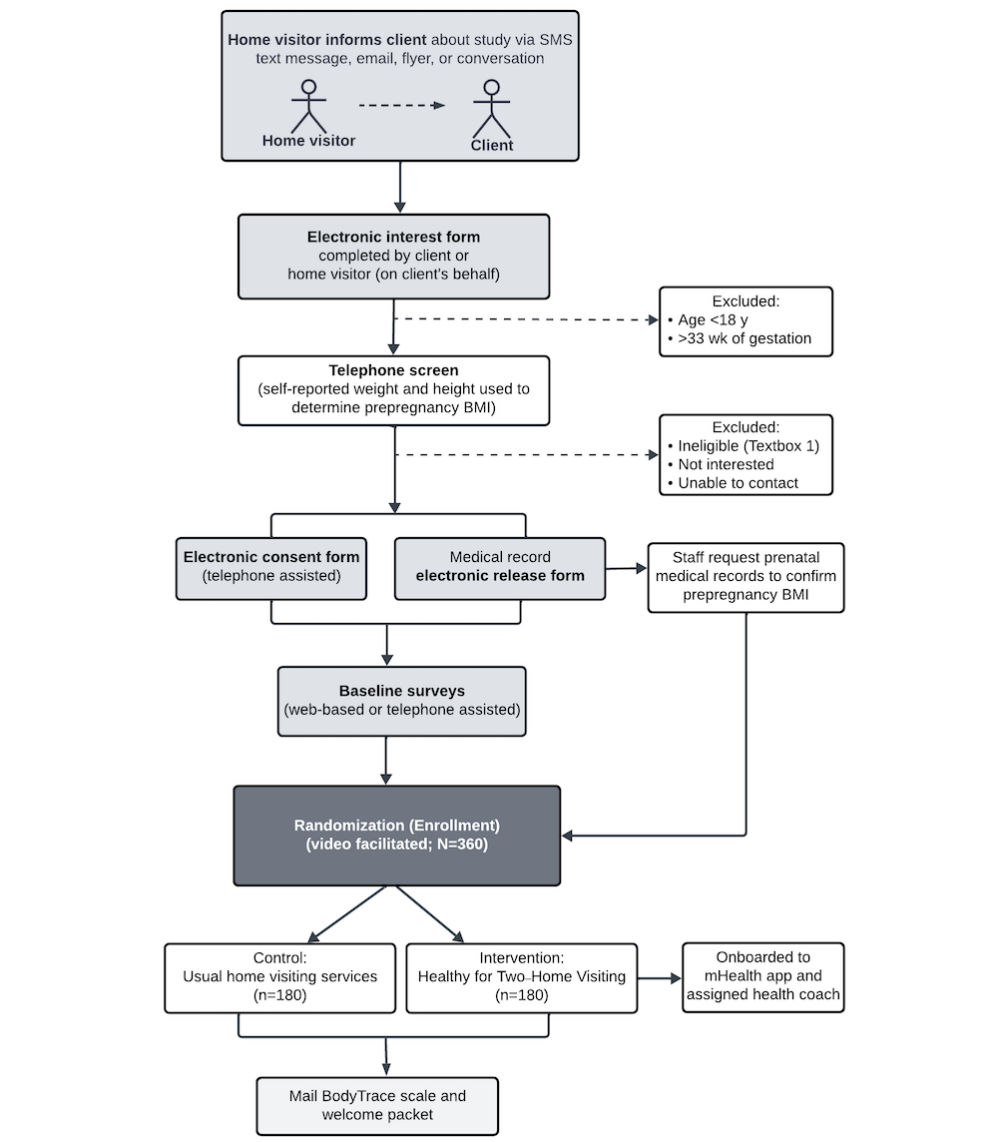
Study design and recruitment procedures. mHealth: mobile health.

With feedback from home visiting program partners (refer to the Application of a Community-Engaged Approach subsection), we designed the role of home visitors to be low touch and aligned with the procedures they already use in their program and visits. Figure 2 outlines the study design and recruitment procedures. Home visiting staff inform potentially eligible clients about the study via conversation, email, or SMS text message using a “toolkit” of different materials available in English and Spanish to accommodate program, staff, and client needs and preferences (eg, suggested dialogue, paper flyers or postcards, and an informational video lasting 2-3 min). All recruitment materials include a link and QR code to an “electronic interest form” (to be completed by clients or home visitors on their behalf) that requests basic eligibility information to preemptively exclude clients aged <18 years and >33 weeks of gestation, as well as additional details to facilitate the next steps of the screening process.

Upon receiving a completed “electronic interest form,” research staff reach out to the potential participant via telephone to further assess interest and screen for eligibility. After confirming eligibility, research staff complete a telephone-assisted electronic consent process that includes obtaining a signed authorization for the release of medical records, including prenatal and infant records as well as claims data. After consent is obtained, study staff immediately request prenatal clinic records for height and prepregnancy weight measurements to confirm BMI criteria, and participants complete web-based or telephone-assisted baseline data collection surveys. Once these steps are complete, consented participants meet virtually with staff for a video-facilitated randomization (enrollment) visit. At randomization, participants receive instructions for taking home weight measurements using a study-provided smart scale shipped to their home; intervention participants are oriented to the H42 mHealth app and provided the name of their health coach. In response to home visitors’ interest in the result of each client they refer (ie, ineligible, unable to contact, or enrolled), we provide them with the option to “opt in” to live email updates on referral outcomes.

### Randomization and Blinding

A total of 360 participants will be randomized 1:1 to the H42-HV arm or comparison arm. Randomization is stratified by home visiting program region+primary language served (ie, central Spanish or English, capital Spanish or English, eastern Spanish or English, southern Spanish or English, or western Spanish or English) and BMI (≥30 kg/m^2^ vs 25-29.9 kg/m^2^) and within each stratum using randomly varying block sizes of 2, 4, and 6. The randomization scheme was generated using Stata (version 17.0; StataCorp LLC) and imported into REDCap (Research Electronic Data Capture; version 14.0.31; Vanderbilt University) [56,57]. Assignment remains masked until a participant is randomized. Due to the nature of this lifestyle intervention, participants, home visitors, the intervention team, and the safety monitor will not be blinded to randomization assignment after randomization. Until the end of the trial, all nonintervention study staff and coinvestigators, including the principal investigators and data collectors, will remain blinded, with the exception of the lead biostatistician.

### H42-HV: Intervention Design and Approach

#### Overview

The intervention was adapted from our previously designed and pilot-tested remotely delivered lifestyle intervention (called Healthy for Two/Healthy for You) to limit GWG and PPWR in a racially diverse population with low literacy [33,34]. The person-centered intervention uses a standard behavioral approach to weight management [58], teaching strategies aligned with social cognitive theory, such as self-monitoring, goal setting, and problem-solving [59]. The overarching goal of the H42-HV intervention is for participants to have lower PPWR 6 months after delivery.

#### Intervention Components and Adaptations

##### Overview

We used an iterative approach for translating and adapting intervention content and technologies using feedback from our key stakeholders (refer to the Application of a Community-Engaged Approach subsection). In addition to shifting intervention timing and focus to the postpartum period, we reframed messaging about program goals to achieving “overall health and wellness” versus a “healthy weight.” Consistent early feedback from home visitors suggested that strong internalized weight biases among their clients may impact intervention engagement and acceptability. Weight stigma is pervasive in health care settings, has detrimental impacts on overall health and the use of health care services [60,61], and has more recently been regarded as a social determinant of poor birth outcomes [62]. Textbox 2 summarizes the adapted components of H42-HV.

**Healthy for Two–Home Visiting intervention components.**

**Person-centered health coaching (English or Spanish)**

- 10 total telephone or video meetings (4 pregnancy, 6 postpartum) lasting approximately 30 min using a person-centered approach, plus 2 as-needed “boosters”
- Starts between 20 and 33 wk of gestation and continues through 6 mo post partum
- Coaches have access to a mobile health (mHealth) coaching interface to view participant app engagement and health progress (refer to the H42 mHealth App subsection)

**Self-weighing via a home smart scale**

- Participants self-weigh at least once weekly on a cellular-enabled home smart scale
- Paper and electronic “wellness journal” available to self-monitor diet and exercise

**H42 mHealth app (hosts web-based learning and goal-setting activities, smart scale weight displays, and 2-way participant-coach messaging; promotes engagement via dynamic in-app messages and email reminders)**

- Learning activities: 10 educational modules focused on diet, exercise, social support, stress, mood, and sleep. Learning methods include the following: simple, brief education on core topic; audio quotes from 3 ethnically diverse mothers describing personal challenges or successes and behavioral strategies that help them meet health and wellness goals; 5 simple multiple-choice quiz questions to reinforce key concepts; open-ended free-text questions, ranging from 4-9 total per learning activity, to promote goal-oriented thinking, problem-solving, and identification of barriers and successes.
- Add-on learning: videos and external links covering topics such as breastfeeding, gestational diabetes, and smoking cessation
- Goal setting activity: tool that aids participants in setting their own specific, measurable, achievable, relevant, and time-bound (SMART) goals and rating their progress
- Weight display: real-time view of home smart scale weights with feedback to support goal of returning to prepregnancy weight
- Coach-participant messaging: synchronous communication stream primarily used for scheduling and delivery of individualized intervention content (ie, PDF files, images, etc)
- Home page: personalized summary to facilitate intervention adherence (ie, date and time of upcoming coach meetings, most recent coach message, reminders to weigh) and engagement (ie, seasonal health or wellness “Tip of the week”)
- Coach and coach manager interface: coach interface with dynamic access to participant weight data and engagement with app (ie, SMART goals, free-text entries); coach manager interface with real-time access to participant and group-level data for individualized case management and ongoing support and management of all coaches

##### Person-Centered Health Coaching

The cornerstone of the H42-HV intervention is *health coaching* using an evidence-based person-centered approach [63] aimed at enhancing participants’ intrinsic drive to make health-related behavior changes (diet, exercise, and stress management). Participants complete up to 12 coach meetings (10 planned plus 2 as-needed “boosters”) via video or telephone when they join the study (between 20 and 33 weeks of gestation) through 6 months post partum. Coaches aim to complete 4 meetings during pregnancy and 6 meetings post partum, with flexible cadence to account for varying enrollment dates. The frequency of coach meetings is consistent with similar interventions showing an effect on PPWR [29,33,64] and based on evidence that moderate- (ie, ≥6 contacts) to high-intensity (ie, ≥12 contacts) lifestyle interventions have the greatest effect on GWG [26,65]. Coaches receive enhanced training on weight bias and cultural sensitivity as well as supporting behavioral changes in the context of common social and environmental barriers such as food insecurity and neighborhood safety.

##### Health Behavior Tracking (Self-Weighing via Home Smart Scale)

Participants are instructed to weigh themselves weekly on a *cellular-enabled home smart scale* (Body Trace; BodyTrace, Inc) [66] that transmits live data to the H42 mHealth app and coach interface described in detail in the next subsection. Coaches emphasize that self-weighing is a core tool to assess progress, similar to monitoring one’s exercise minutes and the type and amount of food and drinks consumed. Participants have the option to track and share diet and exercise behaviors with their coach as well as daily ratings of their mood and sleep using a simple paper “wellness journal” or “electronic wellness journal” delivered daily or weekly via SMS text message or email.

##### H42 mHealth App

Our team designed the *web-based mHealth app* (Figure 1) *and coach interface* based on intervention content tested in past trials [33,34]. The H42 mHealth app is accessible via mobile phone and delivers education tailored to a <6th-grade reading level [67-69] via interactive learning activities that provide guidance on making healthy lifestyle changes in the context of common environmental barriers (eg, eating healthy on a budget and low-cost ways to manage stress). Supplemental health topics (eg, breastfeeding, infant health, and depression) are also available because our formative research and work by others suggested that pregnant and postpartum people across races are more likely to use digital health tools that offer credible, perinatal-specific health information beyond nutrition and exercise [70,71]. The mHealth app contains a goal-setting activity, facilitates 2-way participant-coach communication, displays smart scale data, and promotes adherence and engagement via dynamic in-app messages and email reminders (Figure 1). End-user testing of the English and Spanish versions of the app completed in preparation for the trial (the testing involved 3 English-speaking and 3-Spanish speaking parents and 2 bilingual home visitors) generated reactions to app design and images, usability, interactive functionality, cultural appropriateness, and effectiveness. Consistent feedback gathered (and addressed) included preferences for a brighter color palette, more images, less text and fewer numbers, more traditional Latinx food options, larger-sized body types, simpler graphics (ie, bar graph vs line graph), and a stronger representation of family (ie, households with multiple children). If cost is a barrier, the study subsidizes web-based access (eg, by providing data cards).

The *coach/coach manager interface* provides dynamic access to participant smart scale weights and app activity (ie, goals and free-text responses) as well as food and exercise data for those who choose to track these behaviors using the “electronic wellness journal” that syncs data to the interface. The interface additionally serves as a documentation and scheduling tool. A coach manager interface provides individual and aggregate summary data to facilitate regular participant oversight, ongoing support, and the management of coaches and intervention adherence monitoring throughout the study.

### Usual Home Visiting Services Comparison

Participants randomly assigned to the comparison arm receive usual home visiting services per agency guidelines and requirements. In addition, we provide a brief, publicly available educational video on urgent maternal warning signs [72,73]. Private, staff-monitored Facebook groups are offered to disseminate information on healthy pregnancy and allow for community building and retention for both groups (usual home visiting services and H42-HV). Both groups are also provided county-specific resource lists with information on green spaces, food banks, mental health resources, medical centers, and intimate partner violence support. This resource list is available as an electronic map (using Google Maps) and a paper version.

### National Institute on Minority Health and Health Disparities Research Framework Adaptation for the H42-HV Intervention

**Figure 3:**
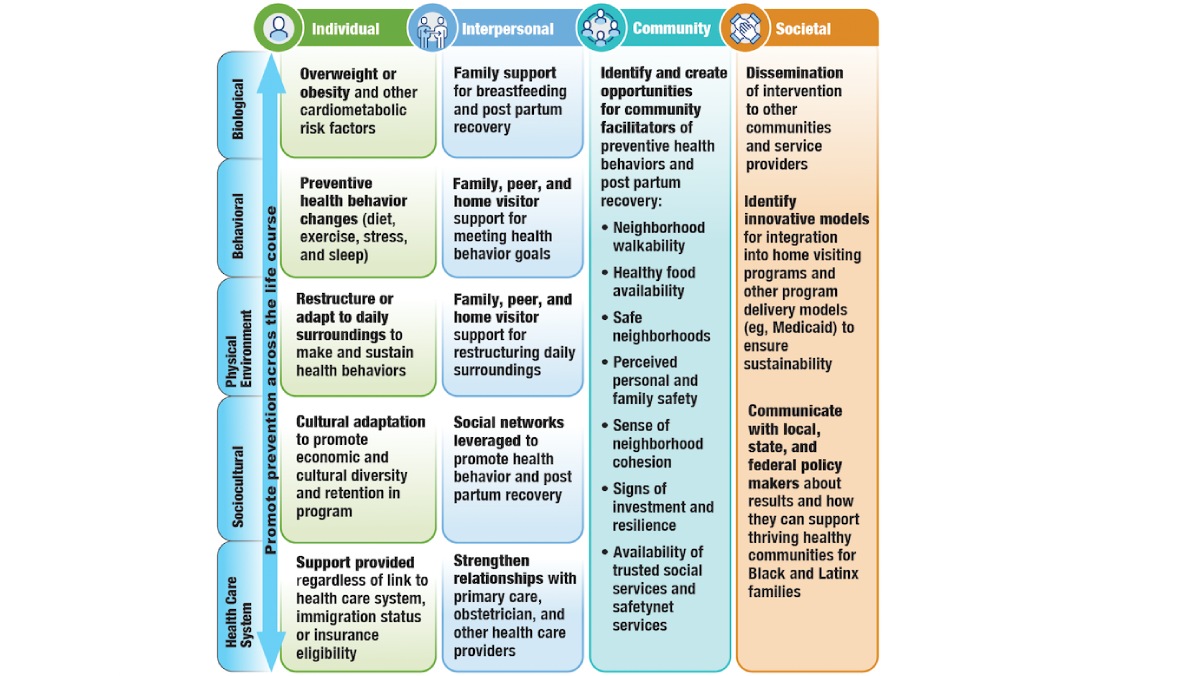
National Institute on Minority Health and Health Disparities research framework adaptation for the Healthy for Two–Home Visiting intervention.

We adapted the National Institute on Minority Health and Health Disparities research framework [74] to depict the multilevel influences (individual, interpersonal, community, and societal levels) that embedding the remote intervention into early home visiting services has on health outcomes and disparities, including the social determinants of health (Figure 3). The H42-HV intervention impacts *individual-level* factors by promoting a healthy lifestyle in women with cardiovascular risk factors, regardless of insurance coverage or health literacy. While coaches provide education and strategies for making healthy changes (ie, adding fruits and vegetables to participants’ diet), home visitors address context-specific barriers (eg, healthy food availability) and leverage context-specific assets (eg, local food banks) to increase success at achieving behavioral goals. At the *interpersonal level*, home visitors provide social support and connect participants with social support networks that promote a healthy lifestyle and provide tools to navigate family or peer norms, while health coaches teach participants effective communication skills to strengthen the support they receive from their existing network (eg, home visitors, health care providers, family members, and peers) and tailor this support toward making healthy changes. The H42-HV intervention addresses *community*- and *societal-level* influences by connecting participants with local resources and promoting parent and infant use of health care services (eg, postpartum care and primary care). Ultimately, the study is designed to promote a holistic approach to reducing cardiometabolic health inequities among birthing people.

### Data Collection and Data Sources

#### Effectiveness Measures and Methods

**Figure 4:**
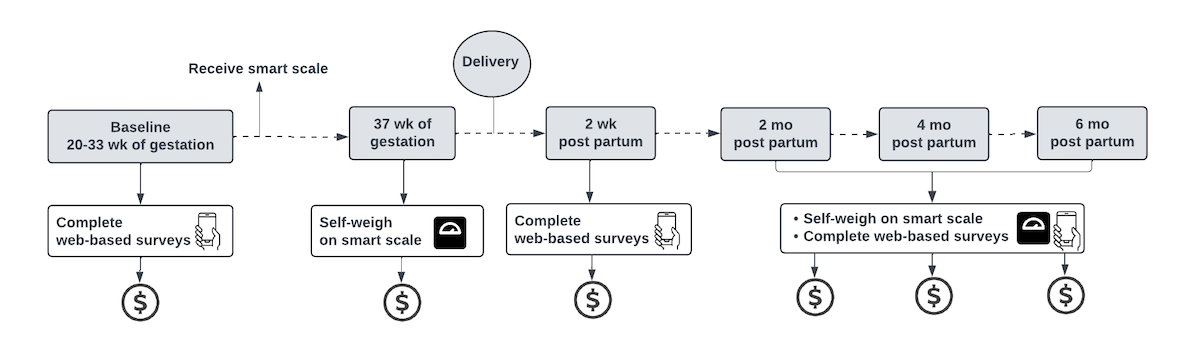
Data collection and retention.

Tables 1 and 2 summarize the methods of measurement and timing aimed at improving access and retention as well as minimizing participant burden (also refer to Figure 4). Early conversations with home visiting program leaders indicated that home visitors would not have time to collect study data; therefore, data collection procedures were designed to not involve home visitors. Data are collected through 4 methods: a cellular-enabled home smart scale, medical record review, web-based surveys via REDCap, and Medicaid claims data.

**Table 1:** Schedule of intervention effectiveness measures: electronic medical record review, smart scale, and Medicaid claims.

Measure		Pregnancy		Post partum			
		Baseline^a^	37 wk	Delivery^b^	2 mo	4 mo	6 mo
Maternal weight and height		Electronic medical record review	Smart scale	—^c^	Smart scale	Smart scale	Smart scale
Labor and delivery discharge summary from outside hospitals		—	—	Electronic medical record review	—	—	—
Infant weight and length from pediatric practices		—	—	Electronic medical record review	—	—	—
Maternal and infant health care use					Medicaid claims	Medicaid claims	Medicaid claims
Home visiting use and safety net services					Medicaid claims	Medicaid claims	Medicaid claims

^a^Baseline window: 20 to 33 wk of gestation.

^b^Delivery through 2 wk post partum.

^c^Not applicable.

**Table 2:** Schedule of intervention effectiveness measures: web-based surveys.

Measure		Pregnancy		Post partum		
		Baseline^a^	Delivery^b^	2 mo	4 mo	6 mo
**Web-based surveys**						
	Demographics and medical history [75-78]	✓	✓^c^			
	Dietary behaviors [79]	✓				✓
	Physical activity [80]	✓		✓		✓
	Depression and anxiety [81]	✓	✓	✓	✓	✓
	Brief Perceived Stress Scale [82]	✓		✓	✓	✓
	Brief Pittsburgh Sleep Quality Index [83]	✓		✓	✓	✓
	Functional Social Support Questionnaire [84]	✓		✓		✓
	Social determinants of health [76,78]	✓				
	Everyday discrimination [85]	✓				
	Tobacco, marijuana, and alcohol (PRAMS^d^) [86]	✓				✓
	Pregnancy intention (PRAMS) [86]	✓				
	Usual source of (maternal) care (PRAMS) [86]	✓				✓
	Experiences with care (PRAMS) [86]		✓			
	Infant care (PRAMS) [86]		✓	✓		
	Postpartum visit attendance and support (PRAMS) [86]				✓	
	Postpartum contraception (PRAMS) [86]				✓	✓
	Breastfeeding intention and practices (PRAMS) [86,87]			✓	✓	✓
	Use of community and safety net services: Supplemental Nutrition Program for Women, Infants, and Children (PRAMS) [86]			✓	✓	✓
	Engagement with home visiting			✓	✓	✓
	Safety survey		✓	✓	✓	✓

^a^Baseline window: 20 to 33 wk of gestation.

^b^Delivery through 2 wk post partum.

^c^Infant race and ethnicity collected at delivery.

^d^PRAMS: Pregnancy Risk Assessment and Monitoring System.

#### Assessment and Verification of Maternal Weight Using a Smart Scale

Smart scale weights are collected at 4 time points: 37 weeks of gestation; and 2, 4, and 6 months post partum (Table 1). Participants are instructed to weigh themselves in light indoor clothes without shoes on their home smart scale (BodyTrace [66]). The smart scale transmits data to the study team via cellular connectivity (no Wi-Fi or cellular plan is required), which is ideal for rural client communities with intermittent Wi-Fi or those with reduced access to cellular data or inconsistent data plans. The BodyTrace smart scale was selected because it demonstrates good concordance with in-person assessments [88,89] and has been used in several large weight management trials [90,91], including those with racially diverse populations with low incomes and literacy levels [92-94]. The scale is mailed to participants’ homes after randomization, and brief SMS text reminders to weigh are sent at each study assessment time point (ie, “Time to step on your scale”). Staff monitor weight data transmitted to the study’s REDCap server in real time and reach out to participants with no weight by the middle of each designated assessment “window,” which ranges from –10 days to +10 days at designated study outcome assessment time points. Staff also monitor battery power and the strength of the cellular connection to assist participants with related issues, as needed. To mitigate the disruption that environmental factors (eg, potential for multiple users or scale displacement) can have on data quality, we programmed a dynamic weight cleaning procedure that requires participants to confirm questionable weights by responding to a 1-question survey sent via SMS text message. For intervention participants, this cleaning procedure ensures real-time accuracy of the weight graphs in the H42 mHealth app, as well as automated reminders, including in-app messages that prompt participants to weigh if a confirmed weight is not available after 7 days. After 14 days, coaches are notified to conduct personalized outreach to remind participants to weigh themselves.

#### Obtaining Medical Records and Abstracting Information on Prepregnancy Weight

Participants consent to pre- and postnatal medical record release for themselves and their infant from before pregnancy through 1 year post partum (Table 1). We use a secure electronic fax system (OpenText Fax; Open Text Corporation) to request medical records from prenatal clinics, offices, and hospitals. “Prepregnancy” weight is defined as the earliest measured weight obtained from medical records up to 18 weeks of gestation; when not available, we use self-reported weight. We also abstract height, parity, and comorbid conditions from medical records.

#### Web-Based Surveys

We used REDCap to build and design web-based surveys using standard instruments selected to minimize participant burden and enable completion at home (Table 2). Collectively, surveys take 10 to 20 minutes to complete, depending on the total number and length of those designated at each time point; staff-led telephone-assisted surveys are available, when preferred.

##### Demographics and Social Determinants of Health

Maternal and infant demographics and social determinants of health are collected using standard questions from the PhenX toolkit [78], the 2020 US Census Informational Questionnaire [75], and the Accountable Health Communities Health-Related Social Needs screening tool [77]. Additional common data elements, using standard and commonly used measures related to participant characteristics and social determinants of health, were incorporated, as required by the National Institutes of Health–Health Equity and Action Network for data harmonization at the National Institutes of Health Multiple Chronic Diseases Disparities Research Consortium [76]. Experiences with chronic, routine discrimination [95] are assessed using the 9-item Everyday Discrimination measure [85], which demonstrates good reliability (Cronbach α=0.88) and is shown to be a strong and consistent predictor of health and well-being [85].

##### Maternal Health Behaviors, Attitudes, and Experiences

Dietary intake is assessed as estimates of servings of fruits and vegetables, added sugars, whole grains, fiber, and calcium using the 26-item Dietary Screener Questionnaire [79], which demonstrates agreement with 24-hour dietary recalls [96]. Exercise frequency and intensity are measured using the 7-item International Physical Activity Questionnaire–Short Form, which has acceptable reliability (pooled ρ=0.76) and some agreement with the accelerometer standard (pooled ρ=0.30) in a diverse sample [80].

Mood is assessed using the 10-item Edinburgh Postpartum Depression Scale for postnatal depression, which illustrates moderately high validity (sensitivity=85%, specificity=77%) and split-half reliability (*r*=0.88) in the original sample of 60 mothers [81]; these results have been confirmed in other validation studies [97]. We measure stress using the 4-item Brief Perceived Stress Scale [98], which is a shortened version of the original 14-item scale [82] and has acceptable psychometric properties [99]. We assess sleep using the 6-item Brief Pittsburgh Sleep Quality Index [83], which shows good internal consistency (Cronbach α=0.79, McDonald ω=0.91) and adequate validity (sensitivity=76%, specificity=77%) in a large population-based sample [83]. Perceived social support is quantified using the 8-item Duke-UNC Functional Social Support Questionnaire [84], which has favorable test-retest reliability (*r*=0.50-0.85) and is significantly correlated with other social support measures [84].

Several questions from the standard and core measures of the Pregnancy Risk Assessment and Monitoring System (PRAMS) [86] assess pregnancy and breastfeeding intentions and practices, contraception, substance use (tobacco, marijuana, and alcohol), and experiences with or use of health care before and after birth.

##### Infant Health, Sources of Care, and Feeding Practices

Infant overall health, feeding, and sources of care are assessed using the PRAMS [86] and Infant Feeding Practices Survey [87]. The use of community and safety net programs (ie, Supplemental Nutrition Program for Women, Infants, and Children) is also measured using the PRAMS [86].

##### Engagement With Home Visiting Services

Engagement with home visiting services and the frequency of contacts with home visitors will be collected at all postpartum time points to assess the “dose” of home visiting during the study.

##### Intervention Satisfaction

Intervention participants complete a satisfaction survey at the end of the study using an adapted survey tool administered and reported on in previous trials [33,34].

#### Medicaid Claims Data

We will request Maryland Medicaid claims data for all consented participants with Medicaid to assess maternal and infant health care use outcomes (ie, attendance at prenatal care visits, postpartum visit, primary care visits, infant visits, and receipt of infant vaccines) via a data use agreement with the Maryland Department of Health (Table 2).

### Implementation Process Measures and Methods

#### Overview

Measures to evaluate the implementation are based on the PRISM framework [45] and domains from the CFIR [46]. Table 3 outlines all implementation outcomes and measures.

**Table 3:** Implementation process measures and methods.

PRISM^a^+CFIR^b^ domains	Implementation process measure	Data collection method (before, during, and after the trial)
Organizational perspectives	Home visiting program perceived usability, adaptability, and relative priority of the intervention	Surveys before and after program orientation; focus groups after the trial
Organizational characteristics (inner setting from the CFIR)	Home visiting program culture, management support and cooperation, systems, training, staffing, and incentives	Home visiting leader surveys before the trial
External environment (outer setting from the CFIR)	Home visiting program regulatory environment (policies and incentives); patient needs and resources	Home visiting leader surveys before the trial; county reports; census and county rankings database
Reach	Total number of clients enrolled out of those screened and eligible; total number of clients enrolled out of new pregnant clients enrolled in the home visiting program	Study recruitment and enrollment data; home visiting program leader surveys after the trial
Implementation (engaging, reflecting, and evaluating process from the CFIR)	Engagement of program leaders in implementation process; qualitative feedback on the progress and quality of the implementation	Coordinating council, formative interviews with home visiting program leaders, focus groups, and research team discussion and reflection throughout the trial
Adoption	Proportion of sites across the state that opt to participate in the study; adoption of training and recruitment procedures; level of involvement supporting intervention participants	Home visiting staff focus groups after the trial; review of study recruitment and enrollment data
Fidelity of the intervention (coach and participant)	Coach adherence to meeting guides and patient-centered approach; participant adherence to intervention components and perceived acceptability	Review of audio-recorded coach meetings during the trial; reports from data management systems; participant acceptability survey after completing the study

^a^PRISM: practical, robust implementation and sustainability model.

^b^CFIR: Consolidated Framework for Implementation Research.

#### Organizational Perspectives

To support state and program leader feedback gathered during the conceptualization phase of the study (refer to the Application of a Community-Engaged Approach subsection), home visitors’ perspectives of the intervention were assessed via survey before and after a 1-hour study staff–led orientation (an overview of study goals, design, and referral procedures) that they received before the trial. They rated the importance of, and the need for, resources to address various health-related topics (eg, nutrition and exercise) with their clients before the training and after they rated intervention acceptability, appropriateness, and feasibility [100]. At the end of the study, we will conduct 2 focus groups with home visitors from participating programs to further explore the perceived usability, acceptability, and adoption of the intervention. Interview guides will be developed using the PRISM framework [45] and include questions assessing facilitators and barriers to implementation.

#### Organizational Characteristics (Inner Setting From the CFIR)

Features of home visiting programs through which the implementation process will proceed and features that may support or impede the programs’ ability to successfully implement the intervention (eg, structure, enrollment, staffing, service modality, and curriculum) were assessed before the trial using a survey completed by home visiting program leaders.

#### External Environment (Outer Setting From the CFIR)

The county-level economic, political, and social contexts within which the home visiting programs reside and which may affect their ability to successfully implement the intervention (eg, social determinants of health, obesity rates, demographics, reimbursements, and health and wellness resources) will be assessed before the trial using a survey completed by home visiting program leaders and publicly available data from county reports, US Census Bureau data [75], and a county rankings database [101].

#### Study Reach

We will quantify study reach as (1) the total number of clients enrolled in the study out of new pregnant clients enrolled in home visiting during the enrollment period and (2) the total number of clients enrolled in the study out of those screened and eligible for the study.

#### Implementation (Engaging, Reflecting, and Evaluating)

We will measure implementation through a combined strategy of gathering feedback from home visiting programs about the progress and quality of the implementation and holding regular debriefings with personnel and team about progress and experience.

#### Adoption of Intervention

We will track the proportion of home visiting sites across the state that opt to participate in the study and assess the level of involvement in study procedures and the intervention via survey and home visitor focus groups after the trial.

#### Fidelity of the Intervention: Coach and Participant Adherence (During and After the Intervention)

We will examine intervention fidelity and its impact on the primary outcome using common procedures applied in multicomponent remote lifestyle intervention trials [102,103]. Health coach fidelity to a participant-centered approach and standard meeting components (eg, reviewing successes and progress as well as setting goals) will be measured using an iterative quality assurance process of sampling and reviewing audio-recorded coach meetings. We will track participant adherence to each component of the intervention (coach meetings, mHealth app, and smart scale use) and intervention acceptability using an end-of-study survey.

### Retention Strategies for Participants

On the basis of our experience with recruiting and retaining pregnant women, we will use several methods to achieve high retention, including rapport building, sending birthday and birth cards, and using email and SMS text message reminders based on each participant’s preferred method of contact. Participants will be provided gift cards after each data collection visit: US $10 at enrollment; US $10 at 37 weeks of gestation; US $15 at 2 weeks post partum; and US $20, US $25, and US $30 at 2, 4, and 6 months post partum, respectively (Figure 4). As participants will be engaged in home visiting and consider the program part of their care, we anticipate low risk for loss to follow-up.

### Methods for Ongoing Home Visitor and Community Engagement

Home visitor engagement will involve monthly recruitment updates shared with sites and site supervisors, raffle incentives, ongoing training opportunities on topics of interest, and brief one-on-one “check-ins” between a study team member and home visitor “site champion” aimed at quickly mitigating concerns or struggles pertaining to study procedures. Community engagement throughout the trial will involve quarterly newsletters to all stakeholders (ie, coordinating council members and state-level leaders), including home visitor and community member “spotlights” and participant success stories. In addition, each home visiting site will receive an annual financial incentive.

### Analytic Approach

#### Sample Size and Power Estimates

With 360 participants, our objective is to determine the minimum detectable difference (MDD) for the primary outcome of PPWR between the 2 study groups. Our assumptions are as follows: a 2-tailed type I error rate of 0.05, a type II error rate of 0.10, and ≥70% follow-up for the main outcome of PPWR at 6 months. On the basis of the past experience [33] and published literature, we anticipate <30% loss to follow-up for 6-month weight measurements, consequential to various forms of dropout (eg, lost to follow-up). With this dropout rate and the assumption that the dropout is consistent with missing at random, we expect to randomize 360 participants (n=180, 50% per arm) to retain an effective sample size of 252 participants (n=126, 50%/arm) for our primary outcome. SDs for the MDD evaluation were informed by previous studies of similar combined diet-exercise lifestyle interventions to limit weight gain in pregnancy and promote postpartum weight loss [26,41,104,105]. Under these considerations, the resulting MDDs range from 2.3 to 3.6 kg with corresponding SDs for PPWR of between 5.5 and 8.8 kg.

#### Main Analytic Model for the Primary Outcome of PPWR

Analysis will follow the intention-to-treat principle. The main analysis will assess the between-group difference in PPWR (the difference between earliest pregnancy weight and weight at 6 mo post partum) using a mixed effects model characterized by a mean model relating the outcome to the predictors and a variance-covariance model addressing variance of all available longitudinal weight outcomes and correlation between outcomes measured over time within individual. The predictors in the mean model will include a group indicator (0 for the comparison arm and 1 for H42-HV) as well as 3 binary indicators for 2-, 4-, and 6-month postpartum visits, respectively, with baseline visit as the reference, and the corresponding group-by-visit interaction terms, adjusting for study sites (region and primary language served) and baseline BMI category used for randomization stratification, as fixed effects. The regression coefficient of the group by 6-month postpartum weight interaction term will estimate the intervention effect on the primary outcome, that is, mean difference in PPWR at 6 months between the intervention and control groups. We will use an unstructured variance-covariance model to allow full flexibility on outcome variances and longitudinal correlations for the repeatedly measured weight data. A model-based 2-tailed *t* test will be used to evaluate the intervention effect and derive the associated 95% CI. The Kenward-Roger approximation will be used to calculate the *df* for the *t* test, with *P*<.05 considered statistically significant [106].

Data from all randomized participants will be used in this analysis, with missing data included using a software-specified missing indicator. The main analysis will assume that outcome data are missing at random and use an observed data likelihood approach implemented through the mixed effects regression model, where baseline characteristics associated with the probability of missing outcome data will be further adjusted for in the mean model. Sensitivity analysis through multiple imputation of missing outcome data under plausible missing-not-at-random scenarios will be conducted to evaluate the robustness of the findings from the main analysis conducted under the missing-at-random assumption.

#### Secondary Outcomes and Additional Analyses

Secondary outcomes include maternal, infant, and organizational process outcomes. For secondary maternal outcomes, available data from all randomized individuals will be included. Between-group differences in GWG (defined as the difference between the weight at 37 weeks of gestation and prepregnancy weight) and infant weights will be assessed using the same mixed effects modeling approach as described for the primary outcome, with separate models for each outcome. Between-group differences in the binary outcomes of diet, exercise, breastfeeding, and women’s wellness measures (depression, sleep, stress, and social support) will be described between the H42-HV and comparison arms using standard cut points for the scales and modeled using logistic regression model–based longitudinal models implemented through a generalized estimating equations approach [107]. The mean models will similarly use the group indicator, visit indicators, and the corresponding group-by-visit interaction terms, adjusting for the variable used to stratify the randomization. Robust variance estimates will be used for statistical inferences to derive 95% CIs for the population-average estimates and corresponding *P* values. Conforming to recommended maternal postpartum care use and well-baby care use over time will separately be modeled using a similar generalized estimating equations approach as described for the longitudinal binary outcomes.

#### Exploratory Analyses for the Heterogeneity of the Intervention Effect

We will explore for potential moderators of intervention effects by conducting subgroup analyses based on baseline survey data (race, ethnicity, home visiting program characteristics, baseline BMI category [overweight or obese], language spoken at home, low English proficiency, income, and education level) and examining effect modification by adding appropriate interaction terms to the primary mixed effects model. We do not expect the intervention effects to vary across subgroups, and we will interpret carefully any observed heterogeneity, or lack thereof, given the exploratory nature of these analyses.

#### Safety Surveillance and Monitoring

For active surveillance, a safety medical officer will oversee the postdelivery review of medical records, including labor and delivery notes and infant discharge summaries. We will administer safety surveys after delivery and at 2, 4, and 6 months post partum to enable tracking of all maternal and infant hospitalizations, emergency department visits, and labor and delivery triage evaluations (Table 2). We have developed protocols to alert the team and manage high levels of depressive symptoms or interpersonal violence (Table 2). The Johns Hopkins Institutional Review Board is required to review all serious safety events. In addition, the study has a sponsor-approved data safety and monitoring plan, and oversight from the Mid-Atlantic Center for Cardiometabolic Health Equity Data and Safety Monitoring Board that meets twice a year to review study progress, intervention adherence, and adverse events (mild, moderate, and severe).

### Ethical Considerations

The protocol received initial approval from the Johns Hopkins Institutional Review Board in June 2022 (IRB00307430) and was determined to be minimal risk. Standard continuing reviews occur yearly; protocol amendments are also reviewed and subsequently updated in the ClinicalTrials.gov registry. During the informed consent process (refer to the Screening and Recruitment subsection), participants are made aware of their right to privacy and confidentiality and are informed that all health information is deidentified or stored on secure servers. They are also advised that they can withdraw from the study at any time without consequence from the research team and medical or home visiting services, and if this occurs, Johns Hopkins may use any data collected before withdrawal. Participants will be provided gift cards after each data collection visit (for details, refer to the Retention Strategies for Participants subsection). In addition, each home visiting site will receive an annual financial incentive.

## Results

This study was funded in June 2021, and recruitment began in April 2023. As of November 2024, we enrolled 90 participants. Data collection to assess the intervention’s effectiveness is expected to end in June 2026. Implementation evaluation is expected to conclude in December 2026.

## Discussion

### Anticipated Findings

We designed this hybrid type I effectiveness-implementation randomized controlled trial to test a remote lifestyle intervention for weight management during pregnancy and post partum in a community-based setting that serves individuals who identify as Latinx and non-Hispanic Black. The goal of this hybrid trial is to evaluate the effectiveness of a newly adapted remote lifestyle intervention (H42-HV) and effectively integrate the intervention into early home visiting services to reduce PPWR. We hypothesize that participants who receive the H42-HV intervention will have a lower mean difference in PPWR at 6 months than control group participants. This would add to the limited evidence supporting the effectiveness of counseling and lifestyle interventions during and after pregnancy in minimizing GWG [25-28] and reducing PPWR [29-32] among racial and ethnic minority groups [32,35]. Furthermore, because few counseling and lifestyle interventions for pregnant and postpartum people have been tested in community-based settings, the use of implementation science methods will enable the gathering of important data about the facilitators and barriers to implementing the intervention in the early home visiting setting and among this population considered vulnerable. Early home visiting programs hold promise to be an ideal setting to integrate lifestyle interventions because of their unique ability to address relevant social and environmental conditions impeding healthy behaviors (eg, access to healthy foods and transportation), as well as support and improve transitions to postpartum care. We anticipate that our study findings will demonstrate feasibility comparable to that reported in another trial of a lifestyle intervention embedded into early home visiting [41,42]. Through the implementation science approach, we will also provide evidence to support policy translation, including the expansion of H42-HV delivery into other US states’ home visiting programs, and into Medicaid Managed Care coaching and case management programs as Medicaid coverage expands into the postpartum period in more states [108].

### Strengths and Limitations

A major strength of the trial’s design is the community-engaged approach, which began during the grant conceptualization and preimplementation phases to inform project design. Community-engaged research approaches have increased dramatically in the last few decades and are linked with statistically positive outcomes and success in recruiting and retaining racially and ethnically diverse populations experiencing marginalization [109-111]. Community-engaged research has many benefits, including ensuring intervention appropriateness, acceptability, and applicability [112-115]; ensuring that study methods and intervention are properly adapted to the population of interest [114,116,117]; and promoting trust, transparency, and bidirectional learning between research teams and stakeholders [112,118,119]. Adopting this approach has already guided key research design decisions, including (1) limiting the primary role of home visitors to the recruitment of study participants to minimize impact on workflow, (2) enrolling participants during mid- to late pregnancy (20-33 wk) to align with client enrollment in home visiting programs, (3) defining the primary outcome as weight retention at 6 months post partum to allow time for increased support during the postpartum period, and (4) focusing study goals and messaging on achieving “overall health and wellness” versus a “healthy weight” to minimize the effects that weight bias internalization may have on recruitment and intervention acceptability. Using remote data collection procedures was another important design consideration (ie, smart scale and access to prenatal medical records), given the transportation barriers of home visiting clients living in rural locations and anticipated challenges they might have in reporting their height and weight to confirm eligibility—an issue that was confirmed soon after study launch. We anticipate that the continued involvement of our coordinating council as well as other methods of community engagement will drive future decisions about the interpretation of data and dissemination of findings.

The iterative process of end-user interviews that informed the design, features, and functionality of the H42 mHealth app was especially valuable for adapting and improving it, including methods for incorporating weight goals and progress (ie, simple, colorful graph versus weight change statistics) and translating the interactive goal-setting activity for Spanish-speaking participants. Comprehensive measures of adherence to coaching, the H42 mHealth app, and the smart scale are a major strength of the study, given the growing complexity of remote lifestyle intervention packages and the critical need to differentiate the effects of unique components [27]. Similarly, access to robust engagement metrics for distinct mHealth app features (ie, interactive goal setting, coach messaging, access to weight data, comprehension quizzes, and educational videos) may build upon the patterns of website engagement characterized by Power et al [120] in a sample of individuals with low-income status who identified as Latinx; of note, in this particular study, website engagement was a strong predictor of weight retention at 6 months post partum.

The design of our study has limitations that could impact the interpretation of the results. First, control participants will have access to a scale for data collection, and regular self-weighing is a key component of behavioral weight management [58]. From a health equity and ethical perspective, we decided that we would refrain from instructing control participants not to weigh themselves outside of data collection and, instead, statistically control for the number of measured weights across the groups. Nonetheless, given the enhanced level of engagement with self-weighing in the intervention group (ie, reminders, ability to view progress on the app, and feedback from the coach), we expect the frequency of weighing in the control group to be significantly lower, and frequency is the strongest known predictor of overall weight change [121]. Another limitation is our limited ability to formally measure and control for the varying levels of support that the home visitors offer clients throughout the trial, which may differentially impact behavior change (eg, addressing access to healthy food and discussing a healthy lifestyle). This lack of control precludes our ability to measure intervention effectiveness for a Latinx and non-Hispanic Black, English- and Spanish-speaking sample considered high risk outside of the context of home visiting. Although home visitors were intentionally removed from intervention delivery, early feedback conveyed a preference among some home visitors to be actively involved, specifically with the ability to access SMART goals (assuming clients’ permission). The differences in home visitor training (ie, nurse vs paraprofessional), curriculum, and the intensity of home visiting models in the trial (ie, frequency of visits ranging from weekly to 2 visits total during the first 6 mo post partum) may also differentially impact client success. We expect qualitative data on intervention adoption captured in focus groups after the trial to enhance our understanding of the potential role home visitors play in moderating intervention effects and will leverage these insights for future trial designs and intervention adaptions.

### Conclusions

There is a critical need to develop effective lifestyle interventions for pregnant and postpartum individuals who identify as Latinx and non-Hispanic Black and experience the greatest risk for adverse pregnancy outcomes. This study has the potential to provide a high-quality assessment of the effectiveness of a remote lifestyle intervention for a Latinx and non-Hispanic Black population considered high risk and highlight facilitators and barriers to its implementation in a grounded service strategy specifically geared toward improving maternal and infant health. We expect the study to yield important findings that aid in refining future lifestyle intervention approaches for pregnant and postpartum people, particularly those who identify as non-Hispanic Black and Latinx, and facilitate scalability in community-based settings, ultimately improving maternal and infant long-term health and promoting health equity.

## Appendix

Multimedia Appendix 1 Peer review report from the National Institute on Minority Health and Health Disparities Special Emphasis Panel - National Institute on Minority Health and Health Disparities - Centers for Multiple Chronic Diseases Associated with Health Disparities: Prevention, Treatment, and Management (P50) ZMD1 MLS (A1) (National Institutes of Health, USA.

## Abbreviations

CFIR: Consolidated Framework for Implementation Research
GWG: gestational weight gain
H42-HV: Healthy for Two–Home Visiting
MDD: minimum detectable difference
mHealth: mobile health
PPWR: postpartum weight retention
PRAMS: Pregnancy Risk Assessment and Monitoring System
PRISM: practical, robust implementation and sustainability model
REDCap: Research Electronic Data Capture
SMART: specific, measurable, achievable, relevant, and time-bound

## Data Availability

The datasets generated and analyzed during this study will be available from the corresponding author on reasonable request.
