# Supplementary material for: Remote Lifestyle Intervention to Reduce Postpartum Weight Retention: Protocol for a Community-Engaged Hybrid Type I Effectiveness-Implementation Randomized Controlled Trial
Source: JMIR Res Protoc. 2025 Jan 7;14:e62847. doi: 10.2196/62847 (PMC11751656; doi:10.2196/62847)
Supplement: Multimedia Appendix 1 [file resprot_v14i1e62847_app1.pdf]

1P50MD017348-01 COOPER, LISA

INCLUSION ACROSS THE LIFESPAN UNACCEPTABLE

RESUME AND SUMMARY OF DISCUSSION: This new application, from Johns Hopkins in collaboration with the University of Maryland Baltimore, and Morgan State University was submitted in response to RFA-MD-21-007, Centers for Multiple Chronic Diseases Associated with Health Disparities: Prevention, Treatment, and Management (P50 Clinical Trial Required). The Investigative Team proposes to establish the Mid-Atlantic Center for Cardiometabolic Health Equity (MACCHE) to address disparities in cardiometabolic disease among socially disadvantaged populations in Maryland.

The Cores are exceptional. The multiple PIs leadership is excellent and the organizational and governance structure are robust. The plans and efforts to reduce the domination of academic and to include community stakeholders as full partners are in place and applicable. A single DSMB with clear plan will monitor the entire program. The development of a standard set of common data elements to be collected, particularly for measures of Social Determinants of Health (SDoH) is a strength. The plans to support the Research Projects are well designed. The strategies for the establishment of the Pilot Project Program are well detailed. There is a detail and clear evaluation plan. The financial commitment of the University for the Pilot Project program is an asset. The didactic trainings for the Early-Stage Investigators to promote ethical, scientifically rigorous, and reproducible research will be good mentoring. The PIs will reach to other Institutions in the region with the intention to recruit women, and persons from underrepresented groups in medicine and biomedical science.

Research Project 1 seeks to use the PIs LINKED-HEARTS Program, a theoretically derived, patient-centered multi-level intervention to address individual and community-level social determinants that affect chronic disease management among Black and Hispanic adults. This program builds on prior work (The RICH LIFE Project) and focuses on addressing structural issues of access and includes a self-measured BP (SMBP) and blood glucose telemonitoring platform, team-based care including a pharmacist and community health worker and provider-level interventions. Two Aims are described., Aim 1, to compare the effects of the LINKED-HEARTS Program versus SMBP alone using a hybrid type I effectiveness-implementation design in a cluster-randomized controlled trial of adults with uncontrolled HTN and either diabetes or CKD. Aim 2, to use the Pragmatic Robust Implementation and Sustainability Model (PRISM) to evaluate the reach, adoption, maintenance of the LINKED-HEARTS program and explore contextual factors associated with adoption and maintenance of the program. The team has expertise to manage the Research Project and a long history of working together. The community input via a Stakeholder Advisory Committee is substantial and the recruitment is possible through existing relationship with the clinics. This efficacy measure is feasible. The Expanded Chronic Care Model is a good conceptual framework including patients, providers, communities and system levels interventions. The use of the Sphygmo Home, a multilingual telemonitoring platform to manage blood pressure and over vital signs is innovative. The community health workers will have flexible roles allowing management of complex needs. The weaknesses include the inclusion of diabetes research without a clear focus. The recruitment methods and eligibility are concerning. The availability of clinician portal in four languages is not well described. Because the intervention is very complex, the determination of which component is responsible for effectiveness could be difficult. The collection of BP at the clinic seems to be unreliable.

Research Project 2 seeks to adapt the Healthy for 2/Healthy for U (H42/H4U), an innovative evidence-based pregnancy/postpartum health coaching intervention that is remotely delivered, into H42/H4U-HV, an integrate health coaching program in early home visiting services, and tailor the intervention for Latinx and Spanish speaking women. Two Aims are described. Aim 1, to expand H42/H4U into six Maryland early home visiting programs by tailoring the intervention for Latinx women and home visiting settings and targeting Black and Latinx pregnant women and infants at highest risk for cardiometabolic

disease (CMD) disparities. Aim 2, to conduct a type 1 hybrid effectiveness-implementation trial to evaluate H42/H4U-HV. Aim 2.A, to compare the effectiveness of H42/H4U-HV integrated into home visiting with usual home visiting services in reducing postpartum weight retention, and Aim 2.B, to employ proven implementation science strategies to evaluate H42/H4U-HV to enable and sustain its integration. The team is qualified to manage the Research Project. The study is supported by preliminary data and has good cultural tailoring. There is good rationale for targeting the Latinx women and Black populations. Community-based principles will be used which will allows the research community participants an actively role in decisions about the research process. The focus on individual, interpersonal and community determinants of obesity is very good. Weight and behavioral goals will be promoted through the COACH Framework a behavioral model guiding coaching calls, behavioral tracking targets and learning activities, an innovative approach for Latinx women. The implementation measures are good. The addition of prevention of CMD in women and their infants is also innovative. Since home visit are already a public health strategy, the sustainability of the multilevel intervention is high. Latinx Advisory Committee will provide language and cultural needs of Latinx women. Some weaknesses are the barriers and facilitators of weight reduction in postpartum women are not defined, which will contribute to difficulties in the determination of the variables contributing to outcomes. The description of how the intervention will address the social determinants is limited. The intervention beside weight lost are not well evaluated and are creating a more complex study. The numbers in recruitment table does not match up the number in the write up. Overall, the intervention is clear but the qualitative assessment is not suitable.

Research Project 3 proposes a randomized trial among populations with low SES, DM, obesity and asymptomatic cardiac dysfunction. Three Amins are described. Aim 1, to use electronic medical record data to compare the association of neighborhood SES with incident HF in patients with diabetes, across urban, rural and suburban settings, and to identify clinical risk factors for HF associated with DM that are more prevalent in persons with low SES. Aim 2, to adapt an evidence-based, pragmatic intervention to improve functional status and risk factors control in persons with low SES, DM, obesity and early cardiac dysfunction, using community-based participatory research (CBPR) and patient-centered outcomes research (PCOR) principles. And Aim 3, to test, in a randomized controlled trial, if a 1-year multilevel intervention of problem solving training, CHW use to enhance social support and health system engagement and use of community facilities to support lifestyle change improves outcomes in those with low SES, DM, obesity and early cardiac dysfunction more than enhanced education and connection with community health programs. The strengths are the team expertise to manage the Research Project, the DECIDE approach which is good and innovative. The community partnership and engagement of patients are robust. The weaknesses are numerous. The conceptual models are not clear and not linked to the proposed study primary outcome. The correlation of heart failure with low SES and diabetes as well as the feasibility of the intervention are not well supported. The results of variable measurements are not being clearly analyzed. Aim 1 will not inform significantly the intervention and will increase the timeline. The exclusion proprieties are not appropriate. Needed preliminary data are not shown. The physical activity data could be too modest to determine cardiorespiratory fitness. The adequate level of retention for final measurements is very unsure. Measurement of VO2 max (maximum (max) rate (V) of oxygen (O_2) your body is able to use during exercise) is not well described. Expertise in physical activity affecting cardiorespiratory fitness is needed. The burden on the participants seems very high. The intervention seems to be too complex.
Overall, The team is excellent and managing this type of work for a long time. The Cores structure is exceptional, efforts to engage underserved researchers and strong empowerment of the community as well as strong connection between Institutions are suitable. The topics and target populations are significant and well justified. The Research Projects are patients centered with delivery in the community settings. The synergy between the Research Projects is clear. However, the potential impacts of the Research Projects on addressing health disparity are uneven. Research Projects 1 and 2 have some easily correctable flaws but Research Project 3 is the main weakness and maybe not

feasible. The add-on to the interventions increase unnecessarily the complexity of the studies and could yield difficulties in determining outcomes. The problems in the Research Projects have been seen as limited guidance from the Cores which could also have an impact on the future Pilot Projects. The application was scored in the excellent range with potential for high impact.

COMPONENT IMPACT SCORES

Cores: 11
Research Project 1: 25
Research Project 2: 33
Research Project 3: 58

DESCRIPTION (provided by applicant): The overarching goal of the Mid-Atlantic Center for Cardiometabolic Health Equity (MACCHE) is to test the effectiveness of comprehensive, integrated, and multi-level evidence-based strategies for improving cardiometabolic health outcomes among socially disadvantaged populations in Maryland, using community- based participatory research and patient-centered outcomes research principles, and translate them into clinical and public health practice and policy. We will leverage the existing infrastructure of the Johns Hopkins Center for Health Equity, which has applied a comprehensive approach to health equity for over 10 years, a partnership with the University of Maryland Baltimore, and collaborations with Morgan State University and other institutions in the region. MACCHE will include 3 separate, but related intervention studies addressing disparities in cardiometabolic disease; 3 integrated cores (Administrative, Investigator Development, and Community Engagement); an Executive Committee; and a Community Advisory Board. Study 1 is a randomized trial comparing the effectiveness of an evidence-based pregnancy/postpartum health coaching/home visiting intervention to usual home visiting services in reducing postpartum weight retention among Black and Latinx women at high risk for cardiometabolic health disparities; Study 2 is a randomized trial examining the effectiveness of a multi-level intervention of problem-solving training, community health worker (CHW) support and partnership with community facilities for enhancing cardiorespiratory fitness in adults with low socioeconomic status, diabetes, obesity and asymptomatic cardiac dysfunction; and Study 3 is a cluster- randomized trial testing the effectiveness of a multi-level intervention linking self-monitored blood pressure (BP) with telemonitoring, team-based care with pharmacists and CHWs, and provider-level interventions compared to enhanced usual care, for improving BP control among socially disadvantaged adults with uncontrolled hypertension plus diabetes or chronic kidney disease. The Administrative Core will carry out the overall administration of the grant, providing infrastructure and support for data harmonization, management and analysis, patient recruitment and retention, and intervention adaptation and implementation. The Investigator Development Core will establish a pilot project program for early-stage investigators and create a mentoring network and community for pilot project awardees, to support innovative research related to chronic disease disparities. The Community Engagement Core will implement a shared governance structure to reinforce stakeholder leadership and ownership; advance, facilitate, and evaluate MACCHE's community- engaged research and investigator development initiatives; and employ community-centered strategies to translate, disseminate, and sustain MACCHE initiatives. The MACCHE will advance the science of cardiometabolic disease disparities and facilitate its translation into clinical and public health practice and policy.

PUBLIC HEALTH RELEVANCE
The overarching goal of the Mid-Atlantic Center for Cardiometabolic Health Equity (MACCHE) is to test the effectiveness of comprehensive, integrated, and multi-level evidence-based strategies for improving cardiometabolic health outcomes among socially disadvantaged populations in Maryland, USA. The

MACCHE will advance the science of cardiometabolic disease disparities and facilitate its translation into clinical and public health practice and policy.

CRITIQUE 1

Significance: 1
Investigator(s): 1
Innovation: 1
Approach: 1
Environment: 1

OVERALL IMPACT:

The applicants propose the Mid-Atlantic Center for Cardiometabolic Health Equity (MACCHE). They focus on cardiovascular disease and its risk factors, hypertension, obesity, chronic kidney disease, and diabetes mellitus in particular. They are proposing multilevel interventions that are, importantly, patient centered with delivery actually in the community, not in the traditional health care environment. They utilize MPI's, with a MPI plan that is well developed. The investigators are a dynamic mixture of incredibly experienced and accomplished senior investigators combined with high accomplished mid-career and junior investigators. In fact, younger investigators are leaders on projects, making them a part of the executive committee for the center. The Community Engagement plan is mature and continues the team's progress toward empowerment of the community as equal partners in research. The presence of health disparities in cardiometabolic health are clearly outlined and there is a sizeable percentage of ethnic minorities and communities that are structurally marginalized. The Investigator Development Core is well structured and builds on past and current success of the institution. The individual projects are all sound, use some common data elements, and test interventions that are designed to reduced disparities. There is connectivity between all parts of the proposal such as every Research Project having a stakeholder advisory committee that evolves from the community engagement core. There is a real connection with the University of Maryland College of Medicine and other community organizations and some FQHCs. The resources at primary institutions are outstanding and will be shared with partner institutions. Some partnering institutions are HBCUs/ MSIs. The institution is investing additional funds into the investigator development core to facilitate the opportunity for additional investigators to benefit from the resources developed in that core.

1. Significance:

Strengths

- It addresses chronic health issues that disproportionately impacts ethnic minorities and those in structurally marginalized communities.
- There are efforts in place to enhance recruitment of investigators from underrepresented in science and medicine into the investigator development core.
- The projects are patient-centered, community engaged, and community centered.

Weaknesses

- None noted.

2. Investigator(s):

Strengths

- The corresponding PI is outstanding.
- The MPI plan is excellent.
- Many of the investigators have worked together for many years and have built outstanding programs that have been a setting for development of young investigators.
- Mid-career and early career investigators are paired with senior investigators throughout the Cores and Research Projects.

Weaknesses

- None noted.

3. Innovation:

Strengths

- The Community Engagement Core's approach is advanced and well on the way to full empowerment of the community in co-partnership with the academic end.
- Younger investigators are given the opportunity to advance their leadership skills in team science.

Weaknesses

- None noted.

4. Approach:

Strengths

- There is clear synergy between all cores and the projects.
- The community stakeholders are prominent in each core and project.
- There is shared leadership and mentorship. Younger investigators are working side by side with senior investigators so that they can develop their leadership skills.
- The MPI plan is well developed.
- The management plan is well developed.
- There is partnership with a neighboring institution for implementing all parts of the program.

Weaknesses

- None noted.

5. Environment:

Strengths

- The structural, people, and intellectual resources are outstanding.
- There are superb core groups for all aspects of implementation of the projects, mentoring, and evaluation.

Weaknesses

- None noted.

Protections for Human Subjects

Acceptable Risks and/or Adequate Protections

- All investigators have completed detailed clinical trials plans. There is a DSMB that will reside in the Admin Core that will monitor all projects.

Data and Safety Monitoring Plan (Applicable for Clinical Trials Only):

Acceptable

- There is a DSMB that will reside in the Admin Core that will monitor all projects.

Inclusion Plans

- Sex/Gender: Distribution justified scientifically
- Race/Ethnicity: Distribution justified scientifically
- For NIH-Defined Phase III trials, Plans for valid design and analysis: Not applicable
- Inclusion/Exclusion Based on Age: Distribution justified scientifically
- All projects with enroll adults only. One study focuses on women who are pregnant.

Resource Sharing Plans

Acceptable

Budget and Period of Support

Recommend as Requested

ADMINISTRATIVE CORE

Strengths

- The corresponding PI has extensive experience in running large research programs.
- There is a MPI plan in place that is excellent and detailed.
- The operating structure is clearly outlined.
- The management plans are clearly outlined.
- It profits from a strong foundation of the Center for Health Equity.
- The Administrative Core houses a single DSMB for the entire program.
- There are common data elements that will be managed in Administrative Core.
- There is an intervention core faculty group that will monitor fidelity of interventions.
- There are clear ties to other Cores and all the Research Projects.

Weaknesses

- None noted.

INVESTIGATOR DEVELOPMENT CORE

Strengths

- They will support 5 – 7 health equity scholars a year.

- The institution has pledged an additional $75K to support scholars.
- They will develop a mentoring network for each scholar that includes a primary research mentor, two career mentors, and one core program faculty mentor. One of the career mentors is proposed to come from another funded P50 programs.
- There are plans to enhance recruitment of scholars from underrepresented in science and medicine groups.
- Review criteria and decisions regarding how pilots are chosen for funding are well developed.

Weaknesses

- It is not clear that investigators from the partnering HBCUs/MSI's will have funding opportunities given the significant disparity in resources available to them compared to those at JHU and the University of Maryland. The investigators may consider guaranteeing at least 2 – 3 pilot awards at investigators at these institutions over the 5 year period.

COMMUNITY ENGAGEMENT CORE

Strengths

- The proposed plan is outstanding and is an evolution of activities over several years and is aspirational in working toward shared leadership.
- The community partners are all greatly empowered and will have impact on all aspects of the program.
- There are stakeholder advisory committees for each project.

Weaknesses

- There is potential for burnout for some of the community advisors given the activities outlined. However, there are plans for 100 community advisors. If that is achieved the burnout issue is less of a concern.

RESEARCH PROJECT 1

Significance-2
Investigators-2
Innovation-1
Approach-2
Environment-1

1. Significance:

Strengths

- It seeks to improve blood pressure in patients with poorly control hypertension and diabetes or CKD by enhancing self-monitoring of blood pressure and community level interventions with CHWs.
- The study will be conducted in FQHCs that provide care to underserved populations, almost all of which are majority ethnic minority with high rates of uncontrolled hypertension.
- It introduces a novel self-monitoring, telemonitoring solution to the community, Sphygmo Home.

Weaknesses

- None noted.

2. Investigator(s):

Strengths

- The investigators are all well prepared and have appropriate experience and training to implement the trial.
- One of the Co-leads is an early career investigator but is partnered with an experience Co-lead and experienced research team.

Weaknesses

- None noted.

3. Innovation:

Strengths

- It introduces a novel self-monitoring, telemonitoring solution to the community, Sphygmo Home.
- The CHW will focus on patients' needs beyond those directly related to blood pressure control.

Weaknesses

- None noted.

4. Approach:

Strengths

- Locating from FQHCs is a strength.
- There will be significant input from the community via the stakeholder advisory committee.
- They use the Expanded Chronic Care Model as a framework.
- Having eligibility to uncontrolled hypertension in patients with diabetes or CKD enhances the impact of a positive result from the trial.
- The inclusion of the flexible CHW role is a strength.
- There are contingencies to provide smart phone for those participants who do not have access to one during the study.
- There are patient, provider, community and system level interventions.

Weaknesses

- Setting eligibility as having most recent BP being > 140 mm Hg systolic may bring in participants who are already controlled and were just not at goal that one day.

5. Environment:

Strengths

- The clinics have the patients and there is already a relationship with those clinics with another study that is the foundation for this study.

- All of the necessary support for design, implementation, monitoring, analysis and dissemination are present and are excellent.

Weaknesses

- None noted.

Study Timeline:
Strengths

- It uses EHR to identify eligible participants.
- The timeline is feasible and detailed.
- The LINKED HEARTS Program is already developed.

Weaknesses

- None noted.

Protections for Human Subjects

Acceptable Risks and/or Adequate Protections

- There is a detailed plan for protections.

Data and Safety Monitoring Plan (Applicable for Clinical Trials Only):

Acceptable

- o There is a central DSMB and the Study leads will monitor also.

Inclusion Plans

- Sex/Gender: Distribution justified scientifically
- Race/Ethnicity: Distribution justified scientifically
- For NIH-Defined Phase III trials, Plans for valid design and analysis: Not applicable
- Inclusion/Exclusion Based on Age: Distribution justified scientifically
- This study will only enroll adults.

Resource Sharing Plans

Acceptable

Budget and Period of Support

Recommend as Requested

RESEARCH PROJECT 2

Significance-1
Investigators-2
Innovation-3
Approach-3

Environment-1

1. Significance:

Strengths

- It targets a health condition that is important and where significant health disparities exist among Latinx and Black women when compared to non-Hispanic white women – maternal health disparities.
- Black and Latinx women are more likely to have pre-pregnancy obesity and excess gestational weight gain putting them at higher risk for cardiometabolic disease in the future.
- It builds upon a telephone based intervention (H42/H4U) that showed good results and now adds home coaching (H42/H4U - HV).

Weaknesses

- None noted.

2. Investigator(s):

Strengths

- There are Co-Leads on the project who are experienced, well trained and well prepared to implement this study.

Weaknesses

- None noted.

3. Innovation:

Strengths

- The program will be culturally tailored to Latinx women using community engaged principles with community stakeholders and patient input.
- The intervention will integrate with social services.

Weaknesses

- None noted.

4. Approach:

Strengths

- The study will be based in six home visiting programs in rural, suburban, and urban MD.
- Bilingual research personnel and coaches will be employed and trained for implementation among Latinx women.
- The COACH framework will be used.
- They will use type 1 hybrid effectiveness – implementation trial format.

Weaknesses

- There is not complete rationale for some of the measures beyond weight change.

5. Environment:

Strengths

- The clinics have the patients and there is already a relationship with those clinics with another study that is the foundation for this study.
- All of the necessary support for design, implementation, monitoring, analysis and dissemination are present and are excellent.

Weaknesses

- None noted.

Study Timeline:

Strengths

- The investigators have experience recruiting pregnant women into trials and have relationships with the clinics from which they will recruit.
- This home visit coaching intervention builds upon a phone based coaching intervention that has been studied so tailoring should be accomplished within the proposed timeline.
- They expect to recruit over 33 months. Each participant is followed for about 9 months. The last months of pregnancy and 6 months post-partum.
- The timeline seems quite feasible.

Weaknesses

- The recruitment table shown does not show recruitment of the N=360 participants they expect. It likely shows the 260 they expect to retain when accounting for dropouts. Nevertheless, this is a discrepancy.

Protections for Human Subjects

Acceptable Risks and/or Adequate Protections

- Only pregnant are being enrolled but the risks to them are minimal. There is a central DSMB for the entire program and there are clear monitoring plans.

Data and Safety Monitoring Plan (Applicable for Clinical Trials Only):

Acceptable

- As noted above.

Inclusion Plans

- Sex/Gender: Distribution justified scientifically
- Race/Ethnicity: Distribution justified scientifically
- For NIH-Defined Phase III trials, Plans for valid design and analysis: Not applicable
- Inclusion/Exclusion Based on Age: Distribution justified scientifically
- They will enroll pregnant adult women so the likely ages are 18 - 50 years.

Resource Sharing Plans

Acceptable

Budget and Period of Support

Recommend as Requested

RESEARCH PROJECT 3

Significance-2
Investigators-3
Innovation-6
Approach-7
Environment-2

1. Significance:

Strengths

- They are focusing on an area where significant health disparities exist, heart failure.
- They are proposing to implement a community based, patient centered intervention targeted to patients with early cardiac dysfunction other metabolic risks like DM and obesity and low SES.

Weaknesses

- Aim 1 does not seem to add much to the study.

2. Investigator(s):

Strengths

- The investigators are strong.
- Highly accomplished mid-career and early career investigators are combined with outstanding senior investigators.

Weaknesses

- None noted.

3. Innovation:

Strengths

- The multilevel intervention of problem solving training, CHWs to enhance social support and use of community facilities is unique and is patient centered and community based.

Weaknesses

- Aim 1 is proposed to provide data that might inform the ultimate design of the trial but it is not sure that it will contribute meaningful information to impact this study.

4. Approach:

Strengths

- Using the proven DECIDE (Decision-Making Education for Choices in Diabetes Everyday) model is a strength.
- The community connections to education and exercise is a strength.
- The use of CHWs to connect to the community and health care system and to enhance participant self-management is strength.
- The community engaged approach to refine the intervention is a strength.

Weaknesses

- It is not clear how the work in Aim 1 informs the work in Aims 2 and 3. If it is thought to be critical to refining the intervention then a pilot may be in order before implementing the full trial.
- The estimates of how many of the participants will come into a site to get the final measurements for outcomes are optimistic. Retention activities will need to be reconsidered given community setting for this study.
- The intervention proposed seems too complex to implement. They should limit the trial to feasibility / pilot and simplify outcome measures.
- There needs to be standardization of how the measures of physical activity will be performed.
- How and where will the VO2 max (maximum (max) rate (V) of oxygen (O_2) your body is able to use during exercise) be measured? Is that feasible?

5. Environment:

Strengths

- There are already relationships and experience in recruiting from the sites proposed.
- The resources for data management, data acquisition, study monitoring, etc. are outstanding.
- The relationship with the community stakeholders is well developed.

Weaknesses

- Will participants be able to get to the community centers is a question.

Study Timeline:

Strengths

- The timeline seems feasible but it is an ambitious recruitment given the focus on low SES participants.
- Recruitment will be split between Johns Hopkins and Washington Co – Meritus Health.

Weaknesses

- If data from Aim 1 is to inform Aim 2 and aim 3 the timeline may be optimistic.

Protections for Human Subjects

Acceptable Risks and/or Adequate Protections

- There are adequate plans for protection in place.

Data and Safety Monitoring Plan (Applicable for Clinical Trials Only):

Acceptable

- A DMSB for the entire program is in place to monitor along with the investigators and institutional IRB.

Inclusion Plans

- Sex/Gender: Distribution justified scientifically
- Race/Ethnicity: Distribution justified scientifically
- For NIH-Defined Phase III trials, Plans for valid design and analysis: Not applicable
- Inclusion/Exclusion Based on Age: Distribution justified scientifically
- Only adults will be included. Since it is an intervention to increase physical activity older adults may not be able to participate.

Resource Sharing Plans

Acceptable

Budget and Period of Support

Recommend as Requested

CRITIQUE 2

Significance: 1
Investigator(s): 3
Innovation: 4
Approach: 4
Environment: 1

OVERALL IMPACT:

The overarching goal of the Mid-Atlantic Center for Cardiometabolic Health Equity (MACCHE) is to test the effectiveness of comprehensive, integrated, and multi-level evidence-based strategies for improving cardiometabolic health outcomes among socially disadvantaged populations in Maryland, using community-based participatory research and patient-centered outcomes research principles, and translate them into clinical and public health practice and policy. The three proposed RCTs of interventions include: Project 1, a multi-level intervention linking self-monitored blood pressure with telemonitoring, team-based care with pharmacists and CHWs, and provider-level interventions vs. enhanced usual care for improving BP control in socially disadvantage adults with uncontrolled hypertension plus diabetes or chronic kidney disease; Project 2, a comparison of the effectiveness of an evidence-based pregnancy/postpartum health coaching/home visiting intervention vs. usual home visiting services in reducing postpartum weight retention in Black and Latinx women at high risk for cardiometabolic health disparities; and Project 3, a multi-level intervention of problem-solving training, community health worker (CHW) support, and partnership with community facilities for enhancing cardiorespiratory fitness vs. enhanced education and connection with community health programs among adults with low SES, diabetes, obesity, and asymptomatic cardiac dysfunction.

The application has many strengths. It focuses on chronic diseases that disproportionately affect populations, including African Americans and Latinx, with health disparities, including hypertension, obesity, diabetes, and heart failure, with an effort to address barriers to reducing those disparities. The three Cores are strong and should facilitate the research and the training of investigators who are post-docs, junior faculty, early-career scientist, and underrepresented in the biomedical workforce. The environment is exceptional for facilitating the objectives of the Center, and there is exceptional institutional support. The main weaknesses of the application mainly centered on the projects. Although Project 1 is likely to have a high impact, Project 2 is expected to have a medium impact, and Project 3 is expected to have a low impact. The concerns include an inadequate focus on the mechanisms underlying intervention effectiveness, questions about the need for Aim 1 in Project 3, and a lack of objective measures of physical activity. Nevertheless, considering the overall strengths and weaknesses of the Center, the proposed work is likely to have a high impact on progress in the field.

1. Significance:

Strengths

- The Research Project will leverage existing infrastructure of the Johns Hopkins Center for Health Equity, a partnership with the University of Maryland, Baltimore, and collaboration with Morgan State University, a historically black institution.
- The Research Project employs a theoretical framework.
- All three interventions are multi-level, addressing the individual level and the interpersonal level.

Weaknesses

- None noted.

2. Investigator(s):

Strengths

- The multiple principal investigators are exceptionally qualified and have complementary skills, training, and expertise.
- Dr. Lisa Cooper has extensive expertise in primary care, health equity research, implementation science, stakeholder engagement, equity and quality measurement, and social determinants of health and experience directing large, multi-faceted research centers.
- Dr. Deidra C. Crews brings additional expertise in nephrology, epidemiology, social drivers of health inequities, lifestyle interventions, health services research, and faculty development and research training program leaders. They will lead the overall project as well as the Administrative Core.
- Dr. Beach, who will co-lead the Investigator Development Core, brings extensive experience directing research training and mentoring pre- and postdoctoral students. In addition, she co-chairs the JHU IRB and the JHU School of Medicine Task Force on Diversity, Equity, and Inclusion in Research. Dr. Purnell is also highly capable, bring experience leading educational and training programs for over 150 public health, nursing, and medical scholars and leading institutional efforts to facilitate training initiatives to promote novel collaborations among communities, universities, and health delivery systems.
- The multiple PI plan is appropriate.

Weaknesses

- None noted.

3. Innovation:

Strengths

- A novel feature of this Research Project is the use of Sphygmo Home, a home BP, blood glucose, and vital signs telemonitoring platform that allows patients to record health parameters via manual entry or Bluetooth transmission. A provider portal allows health professionals to monitor these parameters remotely and securely send instant messages to patients.
- Few studies have tested interventions to improve maternal health outcomes among Latinx women who are underrepresented in behavioral weight loss intervention studies.
- Another innovative feature is the adding of improving maternal and infant CMD to early home visiting programs.
- The plan to apply the DECIDE problem solving approach to heart failure is innovative.

Weaknesses

- There appears to be substantial overlap between the Research Project 2 and an on-going RCT (N=380). The difference is that in the existing RCT the intervention is being tested exclusively in a health care setting excluding women monolingual in Spanish.

4. Approach:

Strengths

- The use of RCT designs in all the studies.
- Generally, the plans for recruitment and retention.
- Generally, preliminary data supports the RCTs.
- Use of power analysis to determine sample size considering attrition and effect size.

Weaknesses

- The interventions have multiple components and if effective, the investigators will not know whether certain components were disproportionately responsible for efficacy.
- The control groups are not attention-matched.
- Lack of objective measures of cardiovascular physical activity behavior as might be captured by activity-trackers.

5. Environment:

Strengths

- The environment is outstanding and should ensure the success of the proposal.

Weaknesses

- None noted.

Protections for Human Subjects

Acceptable Risks and/or Adequate Protections

- The protections are appropriate.

Data and Safety Monitoring Plan (Applicable for Clinical Trials Only):

Acceptable

- A single DSMB will monitor data and safety of all three trials.

Inclusion Plans

- Sex/Gender: Distribution justified scientifically
- Race/Ethnicity: Distribution justified scientifically
- For NIH-Defined Phase III trials, Plans for valid design and analysis: Not applicable
- Inclusion/Exclusion Based on Age: Distribution not justified scientifically
- The exclusion of people under the age of 18 is not explained with a scientific justification.

Resource Sharing Plans

Unacceptable

- The data sharing plan does not adequately address when and how the data (not just the accepted for publication results) will be available for sharing. No mention is made of data-sharing agreements and methods and data documentation for sharing.

Budget and Period of Support

Recommend as Requested

- The budget appears to be appropriate and justified.

ADMINISTRATIVE CORE

Strengths

- The multiple PIs are a strength of administrative core.
- The organizational and governance structure is strong.
- The plan for a single DSMB of external faculty members to review the data and safety of the three trials.
- The plan to establish a website for the project to post calls for applications for the pilot awards and other information about MACCHE.
- The evaluation plan includes short, immediate, and long-term goals and metrics for assessing progress.

Weaknesses

- None noted.

INVESTIGATOR DEVELOPMENT CORE

Strengths

- The leadership of the Core is a strength. Dr. Beach brings extensive experience directing research training and mentoring pre- and postdoctoral students. In addition, she co-chairs the JHU IRB and the JHU School of Medicine Task Force on Diversity, Equity, and Inclusion in

Research. Dr. Purnell is also highly capable, bring experience leading educational and training programs for over 150 public health, nursing, and medical scholars and leading institutional efforts to facilitate training initiatives to promote novel collaborations among communities, universities, and health delivery systems.

- The plan to offer early-stage investigators structured didactic training to promote ethical, scientifically rigorous, and reproducible research, including “Responsible Conduct of Research” and “Scientific Rigor and Reproducibility.”
- The plan for multi-disciplinary mentor teams for the awardees consisting of a primary research mentor, two career development mentors, and a core program faculty is a strength of the Core.
- The experience of the team in outreach to applicants from Historically Black Colleges and Universities and Minority Serving Institutions through Morgan State’s School of Community Health and Policy is a strength.
- The ongoing success in soliciting and awarding grants to community organizations through the Urban Health Institute.
- The well-thought-out plan to review applications and support applicants through the process and to encourage innovation high risk/high reward research.
- There is a firm commitment to the Individual Development Core from the JHU Provost’s Office, which will provide $375,000 in kind ($37,000 annually) to supplement the $1,375,000 allocated in the grant budget.

Weaknesses

- None noted.

COMMUNITY ENGAGEMENT CORE

Strengths

- There are several noteworthy strengths. The plan to employ best practices in community based participatory research to ensure that the perspectives of marginalized communities are embedded into the fabric of MACCHE’s research and training initiatives.
- This will include a shared governance structure that places stakeholder as leaders within the Center and its studies to advance, facilitate, and evaluate the project’s community-engaged research efforts and co-design strategies to translate, disseminate, and sustain MACCHE initiatives.
- Another strength is the plan to create study-specific Stakeholder Advisory Committees to facilitate stakeholder-driven strategic decision-making, guidance, and leadership throughout the life of each study.
- Another strength is the effort to reduce academy hegemony, the propensity of researchers to control the level of influence exerted by stakeholders, to allow stakeholders to participate as full partners.
- Plans are in place to address challenges in dismantling academic hegemony such as time constraints of stakeholders by employing virtual collaboration tools and rotating regular meetings among partner locations.

Weaknesses

- None noted.

RESEARCH PROJECT 1

Significance-1
Investigators-1
Innovation-1
Approach-2
Environment-1

1. Significance:

Strengths

- The plan to use a multilevel, multicomponent intervention has the potential to address barriers to reduce BP and disparities affecting African Americans.

Weaknesses

- None noted.

2. Investigator(s):

Strengths

- The PI, Dr. Commodore-Mensah, is well suited to lead this project. She has considerable experience leading community-engaged and clinical research addressing cardiovascular disease risk in Black populations. Dr. Himmelfarb brings complementary expertise in community and stakeholder-engaged research and policy work.

Weaknesses

- None noted.

3. Innovation:

Strengths

- A novel feature of this project is the use of Sphygmo Home, a home BP, blood glucose, and vital signs telemonitoring platform that allows patients to record health parameters via manual entry or Bluetooth transmission. A provider portal allows health professionals to monitor these parameters remotely and securely send instant messages to patients.

Weaknesses

- None noted.

4. Approach:

Strengths

- The study is guided by a conceptual framework, The Expanded Chronic Care Model.
- The study will use a cluster-RCT design to avoid contamination between study arms.
- The planned data analysis appropriately adjusts for clustering among patients within clinics.
- The plan to evaluate implementation outcomes in addition to efficacy outcomes is a strength of the approach.

- Power analysis is presented justifying the proposed sample size considering attrition and effect size.
- The applicant provides evidence of the feasibility of recruitment, considering barriers and ways to surmount them.
- The plans to reduce attrition are excellent.

Weaknesses

- The intervention has multiple components and if it is effective the investigators will not know whether certain components were disproportionately responsible for efficacy.

5. Environment:

Strengths

- The environment supporting the research is excellent.

Weaknesses

- None noted.

Study Timeline:

Strengths

- A detailed study timeline, including recruitment and retention, is included.

Weaknesses

- None noted.

Protections for Human Subjects

Acceptable Risks and/or Adequate Protections

- The protections of human subjects are appropriate.

Data and Safety Monitoring Plan (Applicable for Clinical Trials Only):

Acceptable

- A single independent DSMB will monitor the data and safety of all three trials.

Inclusion Plans

- Sex/Gender: Distribution justified scientifically
- Race/Ethnicity: Distribution justified scientifically
- For NIH-Defined Phase III trials, Plans for valid design and analysis: Not applicable
- Inclusion/Exclusion Based on Age: Distribution justified scientifically
- About 60% of participants will be African American or Hispanic and 50% will be women. Adults ≥ 18 years will be eligible to participate.

Resource Sharing Plans

Unacceptable

- The data sharing plan does not adequately address when and how the data (not just the accepted for publication results) will be available for sharing. No mention is made of data-sharing agreements and methods and data documentation for sharing.

Budget and Period of Support

Recommend as Requested

- The budget appears to be appropriate.

RESEARCH PROJECT 2

Significance-4
Investigators-1
Innovation-4
Approach-5
Environment-1

1. Significance:

Strengths

- The intervention has the potential to address the problem of enduing obesity in Latinx women by intervening during a “teachable moment” pregnancy to reduce risk of cardiometabolic disease (CMD).
- The intervention has the potential to be sustained in the setting since home visiting is already a commonly used evidence-based public health strategy. Moreover, the plan is to partner with organizations already delivering home visits.

Weaknesses

- The significance section does not clarify the barriers to and facilitators of postpartum weight reduction. Most important, there is no conceptual framework that specifies the determinants and mechanisms of the intervention.

2. Investigator(s):

Strengths

- The team has experience developing and testing health coaching interventions for prenatal care settings and culturally adapting interventions.
- Dr. Bower has extensive experience with early home visiting programs.
- Drs. Alvarez and Bennett have experience culturally adapting interventions for Latinx women with low English language proficiency.

Weaknesses

- None noted.

3. Innovation:

Strengths

- Few studies have tested interventions to improve maternal health outcomes among Latinx women who are underrepresented in behavioral weight loss intervention studies.
- Another innovative feature is the addition of improving maternal and infant CMD to early home visiting programs.

Weaknesses

- There appears to be substantial overlap between the proposed study and an on-going RCT (N=380). The difference is that in the existing RCT the intervention is being tested exclusively in a health care setting excluding women monolingual in Spanish.

4. Approach:

Strengths

- There is preliminary evidence suggesting the feasibility and acceptability of the H42/H4U health coaching intervention, making it worthy of testing in a larger R01 type RCT.
- The project uses an adapted NIMHD research framework for the proposed H42/H4U intervention. The intervention is multilevel, addressing individual-level behavior change and engaging interpersonal- and community-level determinants.
- The Research Project will include a Latinx Advisory Committee to provide client/community perspective on adaptation of the intervention to meet language and cultural needs of Latinx women.
- The study will be conducted in a diversity of sites, recruiting from three central region urban home visiting programs, one western region rural program, and two capitol region suburban programs through existing partnerships. Within sites, participants will be randomized to the two arms.
- They address the feasibility of recruitment of 360 women over 36 months and retention of participants is addressed.
- The plan to assess Medicaid claims data as an outcome is a strength.
- The proposed implementation process measures are well justified.
- Sample size is justified based on statistical power.
- The study uses a multisite RCT design.

Weaknesses

- There is no plan to analyze the mediation of intervention efficacy and identify the variables that contribute to positive outcomes and those that do not. In other words, we will not know why the intervention works.
- The control group is not attention-matched; thus, it will not include weekly phone calls for 7 weeks and biweekly calls through 6 months postpartum.
- The intervention is multifaceted; so, we will not know which part (or parts) of the intervention accounts for any observed outcome.
- The description of the intervention did not adequately address the following questions regarding the interpersonal and community level determinants: How do home visitors support communication with family/peers/co-workers and health care providers? How do they provide social support and connect participants with other social support networks that promote healthy lifestyles? How do they help with creative strategies for overcoming barriers presented by physical environment and provide referrals to additional community resources?

- How will participants track weight on Fitbit? Will the BodyTrace scale communicate with Fitbit?
- A minor issue: The rationale for using only two focus groups of home visitors to assess usability, acceptability, and adoption of the intervention, reactions to training; barriers, facilitators, and effectiveness of the home coach-home visitor relationship rather than an individual interviews or surveys is not provided. Focus group discussion can be dominated by one or two vocal participants and can be biased by self-presentation concerns. Individual interviews may provide more informative and independent sources of information particularly when the alternative is only two focus groups.

5. Environment:

Strengths

- The scientific environment is excellent.

Weaknesses

- None noted.

Study Timeline:

Strengths

- A detailed timeline is presented that includes recruitment goals

Weaknesses

- None noted.

Protections for Human Subjects

Acceptable Risks and/or Adequate Protections

- The human subjects protections are appropriate.

Data and Safety Monitoring Plan (Applicable for Clinical Trials Only):

Acceptable

- o A single independent DSMB will monitor the data and safety of all three trials.

Inclusion Plans

- Sex/Gender: Distribution justified scientifically
- Race/Ethnicity: Distribution justified scientifically
- For NIH-Defined Phase III trials, Plans for valid design and analysis:
- Inclusion/Exclusion Based on Age: Distribution not justified scientifically
- The study includes Latinx and Black women who are pregnant: 60% will be Black, 30% Latinx, and 10% white. No scientific rationale is provided for excluding people under the age of 18.

Resource Sharing Plans

Unacceptable

- The data sharing plan does not adequately address when and how the data (not just the accepted for publication results) will be available for sharing. No mention is made of data-sharing agreements and methods and data documentation for sharing.

Budget and Period of Support

Recommend as Requested

- The budget appears to be justified.

RESEARCH PROJECT 3

Significance-4
Investigators-7
Innovation-5
Approach-7
Environment-1

1. Significance:

Strengths

- The proposed research will address disparities in heart failure among low-SES people living with diabetes.
- The application indicates that NIMHD Research Framework is being utilized.
- The application indicates the DECIDE approach will be employed.

Weaknesses

- However, the conceptual models are not tied to the proposed study primary outcome. Therefore, the study lacks a clear and compelling multilevel, multidomain conceptual model that specifies the determinants and mechanisms of action of the intervention on the primary outcome.

2. Investigator(s):

Strengths

- The team has ample experience leading trials to address disparities over 20 years that will inform the proposed work.
- The team includes expertise in cardiometabolic risk and heart failure development.

Weaknesses

- The team has a weakness. The team appears to lack expertise in strategies to increase health enhancing physical activity likely to affect cardiorespiratory fitness.

3. Innovation:

Strengths

- The plan to apply the DECIDE problem solving approach to heart failure is innovative.

Weaknesses

- Aim 1 is not innovative. The investigators have not shown the need to conduct a study to identify prospective correlates of incident heart failure among low SES people with diabetes. They did not show there is a lack of evidence on whether elevated HbA1c, obesity, and hypertension, for example, are related to increased incident heart failure.

4. Approach:

Strengths

- The approach to adapting the intervention is a strength. It will involve input from community organizations, patients, and stakeholders.
- The study employs an RCT design.
- There is a plan to improve recruitment and retention.

Weaknesses

- The conceptual models are not tied to the proposed study primary outcome. Therefore, the study lacks a clear and compelling multilevel, multidomain conceptual model that specifies the determinants and mechanisms of action of the intervention on the primary outcome.
- The need for the observational study to address Aim 1 is not well documented.
- The proposal does not exclude people who are already getting sufficient exercise and are physically fit. These people may not need the intervention; moreover, it may be extremely difficult to raise their level of cardiorespiratory fitness.
- The proposal does not mention preliminary data suggesting the DECIDE problem solving approach increases physical activity or most important cardiorespiratory fitness.
- The study does not include objective measures of increased cardiovascular physical activity as might be captured by an activity-tracking device; therefore, if the intervention is not efficacious the study will not have intermediate outcomes that can shed light on this result. Perhaps, people did not engage in sufficient moderate-to-vigorous exercise.
- Generally, the intervention seems to have too little focus on physical activity to expect significant changes in exercise related outcomes like cardiorespiratory fitness.

5. Environment:

Strengths

- The research environment is excellent.

Weaknesses

- None noted.

Study Timeline:

Strengths

- A detailed timeline includes time to enroll participants.

Weaknesses

- None noted.

Protections for Human Subjects

Acceptable Risks and/or Adequate Protections

- The human subjects protections are appropriate.

Data and Safety Monitoring Plan (Applicable for Clinical Trials Only):

Acceptable

- A single DSMB will monitor the data and safety of all three trials.

Inclusion Plans

- Sex/Gender: Distribution justified scientifically
- Race/Ethnicity: Distribution justified scientifically
- For NIH-Defined Phase III trials, Plans for valid design and analysis: Not applicable
- Inclusion/Exclusion Based on Age: Distribution justified scientifically
- The research will include men and women, African Americans, Latinos, whites age 18 or older.

Resource Sharing Plans

Unacceptable

- The data sharing plans do not provide the required information about sharing.

Budget and Period of Support

Recommend as Requested

- The budget appears to be appropriate.

CRITIQUE 3

Significance: 1
Investigator(s): 1
Innovation: 2
Approach: 2
Environment: 1

OVERALL IMPACT:

This thoughtful, incredibly well-written and organized proposal’s overall goal is to test the effectiveness of multi-level strategies to improve cardiometabolic outcomes in disadvantaged populations in Maryland and build a new cohort of diverse chronic disease health equity researchers. The diverse MPIs and core leaders are outstanding, have a history of partnership and bring together key institutions in the area. Cores are absolutely top notch and build concrete infrastructure to support and evaluate all center activities efficiently and effectively. Engagement is substantive and valued. Regarding Research Projects, there are some concerns about additional, non-key interventions and evaluations that may overly complicate the first two interventions, and that there are no descriptions of those responsible for recruitment and retention in justifications, so it is not clear who will do the work. The third project has

more significant weaknesses and may not be feasible. The disparity between the cores and the projects leads to some concerns that the outstanding cores have not had adequate influence on projects and may not adequately influence them, or pilots, in the future.

1. Significance:

Strengths

- There is a clear thematic focus, rationale and high likelihood the Center will meet its stated objectives of reducing disparities, expanding a diverse workforce and facilitating equitable stakeholder collaborations.
- There is demonstrated need for the trials proposed, pilot data, and endpoints that if met could lead to changes in policies and practices.

Weaknesses

- Diabetes in Research Project 1 and both diabetes and hypertension in Research Project 2 seem to be only peripherally relevant, and perhaps distracting. While it would be good to have focuses on multiple chronic diseases in these projects, in these cases it is more a weakness than a strength.

2. Investigator(s):

Strengths

- Leaders of the grant and its cores and projects are stellar.
- The team are diverse- racially, ethnically and professionally, and are poised to holistically serve the needs of center members and build successful structures and research projects.
- The MPI plans are appropriate and detailed.
- Letters of support show sincere commitment.

Weaknesses

- No significant weaknesses.

3. Innovation:

Strengths

- The key innovation is how the center harmonizes diverse individuals, expertise and operations to serve the larger goals of the center.

Weaknesses

- No significant weaknesses.

4. Approach:

Strengths

- The cores are the key strength. The team has a broad view of what needs to be in a Center- an Administrative Core that houses key people and key activities to serve the center, such as analysts and recruitment experts and formation of a supportive trainee community. Simultaneously, there is great attention to detail in the work- i.e., concrete engagement both

across the Center and for each Research Project, and specific mentoring plans for pilot recipients.
- Research Projects are well organized with sincere attention to rationale, approach, needs of participants and their potential barriers, sustainability and thorough engagement of community, clinical and administrative stakeholders.

Weaknesses
- There are some flaws in the Research Projects outlined within them. Two of three research projects have no listed personnel to conduct recruitment, retention and data collection, for example. The third project has critical weaknesses.
- Much of the detail needed to evaluate proposals was not in the 12 pages, but rather in the supplemental pages.

5. Environment:

Strengths
- Supportive environment for all activities.

Weaknesses
- No significant weaknesses.

Protections for Human Subjects

Acceptable Risks and/or Adequate Protections

Data and Safety Monitoring Plan (Applicable for Clinical Trials Only):

Inclusion Plans
- Sex/Gender: Distribution justified scientifically
- Race/Ethnicity: Distribution justified scientifically
- For NIH-Defined Phase III trials, Plans for valid design and analysis: Scientifically acceptable
- Inclusion/Exclusion Based on Age: Distribution justified scientifically

Resource Sharing Plans

Acceptable

Budget and Period of Support

Recommend as Requested

ADMINISTRATIVE CORE

Strengths
- Led by the MPIs, it has wide expertise, and houses key resources, including for economy of scale, oversight and coordination, including promoting common data elements.
- It houses important activities, including DSMB, seminar series.

- It provides advice and consultation for all aspects of study design, has intervention development, recruitment and retention, biostatistical, mixed methods expertise, a full-time data analyst and data manager, substantive community participation, and people dedicated to center coordination and communications. Resources clearly will maximize scientific rigor and productivity.
- There is a detailed evaluation plan, and all cores and projects submit monthly reports for monitoring.

Weaknesses

- No weaknesses. The core is outstanding.

INVESTIGATOR DEVELOPMENT CORE

Strengths

- The Pilot Project program is accompanied by enhanced mentoring, including a health equity scholars program with three mentors/scholar, required didactics and community building.
- There is a focus on researcher diversity and fostering multi-disciplinary collaboration, including multiple local universities in the region and multiple professions.
- There are matching funds from the university for Pilot Projects.
- There will be a common focus on chronic disease disparities.
- The core team include diverse faculty and community experts.
- There are concrete strategies to solicit projects, diverse scholars, provide technical assistance to applicants, including helping garner design/analytic support, and good peer review and selection process.

Weaknesses

- No significant weaknesses. It is an outstanding core.

COMMUNITY ENGAGEMENT AND DISSEMINATION CORE

Strengths

- Co-led by academic and community partners.
- There is a focus on bidirectional learning, and community are not mere advisors.
- The team are field leaders.
- They are building shared governance that places stakeholders as leads in the center and studies with center-wide and project-specific engagement.
- There are evaluation and sustainability plans, using human-centered design.
- Community letters of support demonstrate true partnership and commitment and community partners are appropriately compensated.

Weaknesses

- An outstanding core with no significant weaknesses.

RESEARCH PROJECT 1

Significance-3

Investigators-2
Innovation-2
Approach-4
Environment-2

1. Significance:

Strengths

- This study compares effect of program vs self-measured BP monitoring in improving BP control and the factors linked with adoption and maintenance of the program.
- It is a multi-level, multisite study with key players needed for success well-engaged and have a history of research collaborations.

Weaknesses

- Nearly all devices, training and attention is paid to BP control. Adding diabetes control makes sense to focus on multiple chronic diseases, but there is too little emphasis to have confidence that this is a feasible Aim.

2. Investigator(s):

Strengths

- Led by nurse-researchers who are early stage investigators, with guidance by more seasoned investigators.

Weaknesses

- No significant weaknesses.

3. Innovation:

Strength

- Multilingual telemonitoring and use of scalable, cost-efficient telehealth platform.
- Dissemination strategy to achieve buy-in and policy change.

Weaknesses

- No significant weaknesses.

4. Approach:

Strengths

- Multi-level intervention with focus on telehealth equity, team-based care and support for structural challenges- broadband, BP cuffs, tech navigation, smartphones.
- Champions for trouble shooting/tech, integration into workflow and recruitment are included at each clinical sites.
- Using Pragmatic Robust Implementation and Sustainability Model (PRISM) to evaluate factors associated with adoption/maintenance of the program.
- Well thought out inclusion/exclusion (i.e., large upper arm circumference).
- Good stakeholder engagement and use of Administrative Core resources (i.e. for training)
- Measures are clearly outlined.

Weaknesses

- Primary outcome BP- not clear how it will be ascertained and by who (no budgeted or described recruiters).
- No mention of who will determine eligibility (i.e. need bicep size) and collect survey and other data, when and how.
- Diabetes seems an afterthought- none of the strengths of the hypertension focus (provision of glucometer, training, CHW focus, data linkage) are described. Not all patients will have diabetes, no exclusion if in DM study). This unnecessarily complicates the study.
- There are no budgeted or described staff for recruitment to supplement the primary approach (EHR messages, which will lead to challenges for those who do not use portals), or to schedule and complete study visits. This leads to concerns about recruitment and significant concerns about retention, especially for 12-and 24-month data collection.
- No description other than in innovation about four languages- i.e., which ones, materials development, is app in all the languages, staff speaking languages,…

5. Environment:

Strengths

- Very appropriate, great engagement with all partners.

Weaknesses

- No significant weaknesses.

Study Timeline:

Strengths

- Appropriate.

Weaknesses

- No significant weaknesses.

Protections for Human Subjects

Acceptable Risks and/or Adequate Protections

Data and Safety Monitoring Plan (Applicable for Clinical Trials Only):

Acceptable

Inclusion Plans

- Sex/Gender: Distribution justified scientifically
- Race/Ethnicity: Distribution justified scientifically
- For NIH-Defined Phase III trials, Plans for valid design and analysis: Scientifically acceptable
- Inclusion/Exclusion Based on Age: Distribution justified scientifically

Resource Sharing Plans

Acceptable

Budget and Period of Support

Recommend as Requested

RESEARCH PROJECT 2

Significance-2
Investigators-2
Innovation-2
Approach-3
Environment-2

1. Significance:

Strengths

- The program assesses impact of expands health coaching into early home visit programs for Black and Latins pregnant women on post-partum weight retention.
- Focus on individual, interpersonal and community determinants of obesity
- Builds on exiting platforms the team developed using both cellphones for virtual content and early post-partum home visits.
- Good sustainability plan.

Weaknesses

- There are extra interventions and assessments that do not appear to be central to the study or part of the analyses (infant weight, teaching about diabetes and hypertension). These could make the study more diffuse, complicate the roles of those delivering the intervention and needlessly add time for participants to submit data.

2. Investigator(s):

Strengths

- Well suited for this grant.
- Community partners well engaged.

Weaknesses

- No significant weaknesses.

3. Innovation:

Strengths

- Diversifying individuals in weight loss studies.
- Focusing on under- attended time/place- post-partum home visits, and using to focus on the mother, not just on her children.

Weaknesses

- No significant weaknesses.

4. Approach:

Strengths

- Good preliminary data- expanding to include Spanish-speaking women and home visits.
- Good cultural/linguistic tailoring and community engagement and building network of early home visiting programs.
- Use of body trace scale to remotely measure weight.
- Remote consent, monitoring.

Weaknesses

- Extra components of intervention not well described not intervention targets, and may distract from study's primary goals (i.e., infant weight via maternal self-report).
- Sample size driven by the number of participants can can be enrolled, is not scientifically correct. This is concerning, and the clinical significance of a 2 kg difference between the 2 groups should be justified.
- Should also have BMI so can determine if individuals are overweight or obese for risk stratification and to see if there are categorical changes.
- Some focus on diabetes, and hypertension, but glucose/A1c and BP are not measured, so intervention components added but not evaluated.

5. Environment:

Strengths

- Very appropriate.

Weaknesses

- No significant weaknesses.

Study Timeline:

Strengths

- Well thought out.

Weaknesses

- No significant weaknesses.

Protections for Human Subjects

Acceptable Risks and/or Adequate Protections

Data and Safety Monitoring Plan (Applicable for Clinical Trials Only):

Acceptable

Inclusion Plans

- Sex/Gender: Distribution justified scientifically
- Race/Ethnicity: Distribution justified scientifically
- For NIH-Defined Phase III trials, Plans for valid design and analysis: Scientifically acceptable

- Inclusion/Exclusion Based on Age: Distribution justified scientifically

Resource Sharing Plans

Acceptable

Budget and Period of Support

Recommend as Requested

RESEARCH PROJECT 3

Significance-2
Investigators-2
Innovation-3
Approach-6
Environment-2

1. Significance:

Strengths

- The program determines association of neighborhood SES with heart failure, identify clinical risk factors for HF associated with DM, and adapt an intervention to improve functional status and conduct an RCT of CHW use to enhance social support on cardiorespiratory fitness. Good focus on reducing HF progression.
- Adapting an effective self-management program for other chronic diseases, to prevent symptomatic HF.

Weaknesses

- Aim 1- it does not seem that it will influence later Aims and earlier research by the team seems to provide the information needed. Investigators hypothesize that variables such as an elevated HbA1c, obesity, hypertension and a low frequency of outpatient clinic visits, will be potentially modifiable target variables. They will assess and address these in the intervention without doing the analysis. While social and environmental factors contribute to HF risk, aim 1 is not likely to change risk reduction strategies.
- With a lack of pilot data showing that the team has developed the intervention, or taken any patients through the numerous types of data collection (fitness testing, walk testing, labs, surveys,…) at repeated timepoints, without an estimate of the time this will take, or the burden on patients, it is difficult to have confidence that the study will be completed. Data on recruitment/retention from the team refer to much simpler trials (i.e. primary outcome BP).

2. Investigator(s):

Strengths

- Talented team with all needed members.

Weaknesses

- May need additional clinical trial expertise, given the lack of piloting the intervention or the data collection.

3. Innovation:

Strengths

- Interesting to add exploratory sub-study examining novel markers of cardiac injury and prognosis.

Weaknesses

- No significant weaknesses.

4. Approach:

Strengths

- Well described steps.
- Good engagement.
- Most Aims 1 and 2 are well described.

Weaknesses

- Aim 3- The intervention has not been developed or piloted- the interventions “adapted” are different from this one, and pilot data is needed before implementing the trial.
- There is a long list of measures to be collected. It is not clear how long this will take, whether they have taken any patients through all the steps, have conducted these types of multi-step, intensive data collection protocols in the past (these are much more complicated than a BP or diabetes trial), how patients respond, how scheduling will be done, etc. Thus, we cannot determine if this is feasible, if patients will return, if the data collection and management systems are well developed, and so on.
- Aim 1 results do not seem to be needed for the other Aims, it appears to be a largely unrelated study.

5. Environment:

Strengths

- Appropriate.

Weaknesses

- No significant weaknesses.

Study Timeline:

Strengths

- Many steps are detailed.

Weaknesses

- There is the concern that one year will not be sufficient to develop the intervention, and there is no time for piloting and revising the intervention or the very intensive and extensive data collection.
- The team are being trained and patients recruited while the intervention is still in the development phase.

- The time to recruit is too short (9 months for 300 patients) without knowing how long each recruitment/data collection visit will take.

Protections for Human Subjects

Acceptable Risks and/or Adequate Protections

Data and Safety Monitoring Plan (Applicable for Clinical Trials Only):

Acceptable

Inclusion Plans

- Sex/Gender: Distribution justified scientifically
- Race/Ethnicity: Distribution justified scientifically
- For NIH-Defined Phase III trials, Plans for valid design and analysis: Scientifically acceptable
- Inclusion/Exclusion Based on Age: Distribution justified scientifically

Resource Sharing Plans

Acceptable

Budget and Period of Support

Recommend as Requested

ADMINISTRATIVE CORE
(Description as provided by applicant)
The Administrative Core of the Mid-Atlantic Center for Cardiometabolic Health Equity (MACCHE) will be responsible for the overall administration of the grant, including guiding and coordinating shared data and intervention resources, Community Engagement Core and Investigator Development Core activities and supporting the three research studies. The overarching goal of MACCHE is to test the effectiveness of comprehensive, integrated, and multi-level evidence-based strategies for improving cardiometabolic health outcomes among socially disadvantaged populations, using community-based participatory research and patient-centered outcomes research principles, and translate them into clinical and public health practice. The Administrative Core, constituted of experts in Clinical Medicine, Nursing, Epidemiology, Biostatistics, Behavioral Science, Health Services Research, Implementation Science, and Community-Based Participatory Research, will be responsible for executing that overarching goal and assuring that the “whole [Center] is more than the sum of its parts”. The MACCHE Administrative Core will build upon the strong existing administrative structure in the Johns Hopkins Center for Health Equity, which has used a comprehensive approach to health equity that includes research and translation, education and training, community engagement, and local to global learning since 2010.
Animal Subject Code: N
Human Subject Code: N

INVESTIGATOR DEVELOPMENT CORE
(Description as provided by applicant)

The Mid-Atlantic Center for Cardiometabolic Health Equity (MACCHE) will establish an Investigator Development (ID) Core with two objectives: (1) to promote career development for Early-Stage Investigators establishing research careers focused on health disparities for chronic diseases; and (2) enhancing diversity of the workforce in this area of biomedical research. MACCHE includes a partnership between The Johns Hopkins University (JHU) and University of Maryland Baltimore (UMB) – both of which have Centers for Health Equity and both of which are centers of excellence for biomedical research. To accomplish its objectives, the Investigator Development Core has established three specific aims: Aim #1. To establish a pilot project program for postdoctoral fellows, early career faculty and other Early-Stage Investigators to support innovative research related to chronic disease disparities. The pilot award program will solicit applications from early-stage investigators at JHU, UMB, Morgan State University, community partners, and the surrounding Mid-Atlantic region who have an interest in addressing chronic, cardiometabolic disease disparities. MACCHE's pilot program will work deliberately to promote pilot applications from persons from underrepresented groups in biomedical research, including those who have faced disadvantage, disability, or come from an under-represented racial/ethnic group. After research applications undergo peer review, applications will be selected for funding based on scientific merit as assessed by peer review in combination with strategic considerations as determined by MACCHE's Steering Committee. Aim #2. To create a multidisciplinary mentoring network and community for pilot project awardees (“MACCHE health equity scholars”) inclusive of senior faculty with health equity expertise. With oversight by MACCHE's ID Core, Early-Stage Investigators receiving MACCHE pilot awards will establish mentoring committees including a primary mentor and two co-mentors to provide overall career mentoring in scholar's background discipline. When possible, we will match scholars with a `national' mentor from another one of the P50 programs. In addition, one of our Core program faculty advisors will oversee the scholar's progress in the program. Aim #3. To support Early-Stage Investigators with structured didactic training to promote ethical, scientifically rigorous, and reproducible research to address chronic disease disparities. Pilot grant awardees will be required to participate in didactic training on “Responsible Conduct of Research” and “Scientific Rigor and Reproducibility” offered at one of the parent institutions. In addition, they will be encouraged to participate in other career development opportunities, including courses on Grant Writing, Scholar Skills and Community- Building, and Writing Accountability Groups. By providing pilot research funding in combination with enhanced mentoring, MACCHE's Pilot Award Program will promote innovative multi-disciplinary chronic disease disparities research in the region and also enhance the diversity of the biomedical research workforce.
Animal Subject Code: N
Human Subject Code: N

COMMUNITY ENGAGEMENT CORE
(Description as provided by applicant)
The values and principles underlying community-engaged research have led to its prominence as an essential strategy for conducting health disparities research, particularly in the realm of cardiometabolic conditions. By centering the equitable engagement of all collaborating partners during each stage of the research process, community-engaged research holds promise for supporting the cultural relevance, translation, and dissemination of promising interventions that disrupt the relationships between socially patterned structural factors, and the consequent emergence of health inequities. However, it is not enough to merely consult with or involve stakeholders in the research enterprise. To fully realize the power of community-engaged research, stakeholder partners must be positioned and acknowledged as leaders, co-creators, and co-developers. This requires intentional efforts to amplify stakeholders' influence throughout every phase of the research process through shared leadership and ownership. The Community Engagement Core (CEC) of the proposed Mid-Atlantic Center for Cardiometabolic Health Equity (MACCHE), jointly led by Dr. Lisa A. Cooper, Reverend Debra Hickman, and Dr.

Chidinma A. Ibe, will serve as the MACCHE's focal point for cultivating, organizing, and promoting bi-directional learning and communication between consortium partners and a robust cadre of multisectoral stakeholders. To accomplish this goal, we will employ best-practices in community-based participatory research to ensure that the voices and perspectives of marginalized communities are embedded into the fabric of MACCHE's research and training initiatives. Specifically, we will 1) construct and implement a shared governance structure that places stakeholders as leaders within the Center and its studies; 2) advance, facilitate, and evaluate MACCHE's community-engaged research efforts, and 3) co-design strategies to translate, disseminate, and sustain MACCHE initiatives. Taken together, these efforts will cultivate an environment of collaboration that amplifies the lived experiences and expertise of our partners to facilitate the translation, dissemination, adoption, and sustainability of our interventions and key research findings. This has implications for the success of our research and for advancing health equity in the Mid-Atlantic region.
Animal Subject Code: N
Human Subject Code: Y

RESEARCH PROJECT 1
(Description as provided by applicant)
A Cardiometabolic Health Program LINKED with Clinical-Community Support and Mobile HEAlth TelemonitoRing in Underserved PopulaTionS (LINKED-HEARTS PROGRAM)
Innovation in chronic disease management is urgently needed to effectively control hypertension (HTN) and diabetes, conditions which affect millions of Americans. Uncontrolled HTN and diabetes cause cardiovascular disease, stroke, chronic kidney disease (CKD), and premature death. However, these conditions are poorly controlled despite the availability of effective and affordable therapy. A pressing priority is reducing disparities in the management and control of chronic diseases and making primary care more convenient for underserved populations. Black and Hispanic adults are disproportionately affected by HTN and diabetes than White adults. They also experience more adverse social determinants of health, including a lack of access to reliable transportation and fragmented access to primary care. Team-based care including community health workers and pharmacists are “best practices” in improving HTN and diabetes control. Telehealth has become a cornerstone of efforts to minimize disruptions in primary care and can be enhanced with remote patient monitoring devices. The COVID-19 pandemic has spurred efforts to increase access to timely and appropriate care through re-engineering primary care to be patient-centered and digitally-enabled. Sphygmo Home, a remote patient telemonitoring solution that links with validated blood pressure (BP) and glucose monitoring devices is a promising solution to improve patient's self-management of HTN and diabetes. We have designed the LINKED-HEARTS Program, an innovative, theoretically derived, patient-centered, multi-level intervention to address individual and community-level social determinants that affect chronic disease management. The LINKED-HEARTS Program focuses on addressing structural issues of access and includes a self-measured BP(SMBP) and blood glucose telemonitoring platform; team-based care including a pharmacist and community health worker and provider-level interventions. Using a hybrid type I effectiveness-implementation design, our proposed specific aims are 1) To compare the effect of the LINKED-HEARTS Program versus SMBP alone in improving BP control (systolic BP<140/90 mm Hg) and improving patient-centered outcomes at 6 and 12 months, in a cluster-randomized controlled trial of adults with uncontrolled HTN and either diabetes or CKD. 2) To use the Pragmatic Robust Implementation and Sustainability Model (PRISM) to evaluate the reach, adoption, maintenance of the LINKED-HEARTS program at 12 and 24 months post-randomization and explore contextual factors that associated with adoption and maintenance of the program. We will enroll 600 adults, clustered in 16 practices including federally qualified healthcare centers. Through early and continued stakeholder engagement with health system leaders, providers, patients, and our community, we seek to close the wide “know-do-gap” and reduce chronic disease disparities. We also propose a

comprehensive dissemination strategy to reach critical audiences and achieve buy-in and policy change.

Animal Subject Code: N
Human Subject Code: Y

RESEARCH PROJECT 2
(Description as provided by applicant)
Effectiveness of an evidence-based health coaching program for reducing cardiometabolic risk among women and infants enrolled in early home visiting services

Black and Latinx women have the highest prevalence of obesity3. Women entering pregnancy with obesity have an excess risk of gestational diabetes, hypertensive disorders, and acute cardiovascular event during labor and delivery, compared to normal weight women4,5. Because pregnant women are motivated to have a healthy baby, pregnancy provides the ideal “teachable moment” to not only reduce adverse pregnancy outcomes, but ultimately prevent long-term CMD in women and their infants. Lifestyle interventions addressing obesity in pregnancy have the potential to break the cycle of obesity and cardiometabolic disease (CMD) for Black and Latinx women8. However, despite evidence of effectiveness, few lifestyle interventions have been tested among Black or Latinx pregnant women or been implemented in community-based settings, where many high risk pregnant and postpartum women access safety-net services. To address this gap, we will leverage our team's experience designing and testing an evidence-based pregnancy/postpartum health coaching intervention that is remotely delivered (phone coaching using motivational interviewing + web-based platform + mobile phone behavioral tracking). Along with our Maryland home visiting partners, we will adapt and implement H42/H4U into the home visiting setting, i.e., H42/H4U-HV and tailor the intervention for Latinx and Spanish speaking women. Early home visiting is an evidence-based public health service strategy found in all 50 states that targets services to high-risk communities to address adverse social determinants of health. Home visitors provide health education, promote positive parenting and early learning, and link families with needed community resources. While home visiting programs don't universally prioritize CMD risk in their services, they are an ideal service-strategy for integration of a healthy lifestyle intervention. We will use a hybrid type 1 effectiveness-implementation randomized control trial to compare the effectiveness of H42/H4U- HV integrated into home visiting compared with usual home visiting services in reducing postpartum weight retention (difference between pre-pregnancy weight and weight at 6 months postpartum) among 360 pregnant and postpartum women. We will also evaluate the implementation of the intervention to enable and sustain integration into home visiting. Health Disparities Impact. A tailored and targeted remotely-delivered health coaching intervention implemented into early home visiting has potential to promote healthy lifestyle behaviors and eliminate disparities in obesity, adverse pregnancy outcomes, and long-term CMD among young high risk Black and Latinx pregnant and postpartum women and their infants. Our research approach allows us to not only establish the effectiveness of H42/H4U-HV but also understand the factors that enable intervention implementation to inform sustainability, further the pathway from evidence translation into practice, and facilitate greater subsequent public health impact.

Animal Subject Code: N
Human Subject Code: Y

RESEARCH PROJECT 3
(Description as provided by applicant)
Understanding and addressing risks of low socioeconomic status and diabetes for heart failure

Heart failure (HF) is associated with high morbidity, mortality and costs, and there is great interest in refining strategies to reduce HF risk. Diabetes (DM) and low socioeconomic status (SES) are each independent risk factors for HF, and both factors together have a synergistic association with incident HF. A major functional consequence of these associations is impaired cardiorespiratory fitness, with

implications for prognosis and quality of life. Additionally, low SES and DM are over-represented among racial and ethnic minorities and therefore a cause of HF disparities. Strategies to address the high HF risk associated with the combination of low SES and DM will require a focus on social determinants of health. Problem-solving training and community health worker (CHW) support are effective in overcoming barriers to care, and improving lifestyle, DM self- management, health system engagement and risk factor control, but they have not yet been applied to addressing HF risk. Prevention efforts would be further informed by understanding geographic disparities in HF risk and elucidating clinical risk factors that might serve as targets for intervention. We therefore propose a randomized trial among 350 persons with low SES, DM, obesity and early cardiac dysfunction, testing the effects of a multi-level intervention of problem-solving training, CHW support and partnership with community facilities to support lifestyle change on fitness, risk factor control, markers of cardiac injury/fibrosis and quality of life. We propose: Aim 1: To use electronic medical record data to a) compare the association of neighborhood SES with incident HF in patients with DM, across urban, rural and suburban settings, and b) to identify modifiable clinical risk factors for HF associated with DM that are more prevalent in persons with low SES. Aim 2: To adapt an evidence-based, pragmatic intervention to improve functional status and risk factor control in persons with low SES, DM, obesity and early cardiac dysfunction, using community-based participatory research (CBPR) and patient-centered outcomes research (PCOR) principles. Aim 3: To test, in a randomized controlled trial, if a 1-year multilevel intervention of problem-solving training, CHW use to enhance social support and health system engagement, and use of community facilities to support lifestyle change improves cardiorespiratory fitness and related outcomes in those with low SES, DM, obesity and early cardiac dysfunction, more than enhanced education and connection with community health programs. This work will elucidate strategies to address HF risk related to low SES and DM, which are key contributors to HF disparities.
Animal Subject Code: N
Human Subject Code: Y

THE FOLLOWING SECTIONS WERE PREPARED BY THE SCIENTIFIC REVIEW OFFICER TO SUMMARIZE THE OUTCOME OF DISCUSSIONS OF THE REVIEW COMMITTEE, OR REVIEWERS' WRITTEN CRITIQUES, ON THE FOLLOWING ISSUES:

PROTECTION OF HUMAN SUBJECTS: ACCEPTABLE

INCLUSION OF WOMEN PLAN: ACCEPTABLE

INCLUSION OF MINORITIES PLAN: ACCEPTABLE

INCLUSION ACROSS THE LIFESPAN: UNACCEPTABLE
Concern Research Project 2: No scientific rationale is provided for excluding people under the age of 18.

DATA SHARING PLAN: CONCERNS
All Research Projects: Publication of results but not the data.

COMMITTEE BUDGET RECOMMENDATIONS: The budget was recommended as requested.

Footnotes for 1 P50 MD017348-01; PI Name: COOPER, LISA A

NIH has modified its policy regarding the receipt of resubmissions (amended applications).See Guide Notice NOT-OD-18-197 at https://grants.nih.gov/grants/guide/notice-files/NOT-OD-18-197.html. The impact/priority score is calculated after discussion of an application by averaging the overall scores (1-9) given by all voting reviewers on the committee and multiplying by 10. The criterion scores are submitted prior to the meeting by the individual reviewers assigned to an application, and are not discussed specifically at the review meeting or calculated into the overall impact score. Some applications also receive a percentile ranking. For details on the review process, see http://grants.nih.gov/grants/peer_review_process.htm#scoring.
